# Metabolic Astrocytic Support with Decanoic Acid Enhances Energy Metabolism in Alzheimer’s Disease Models

**DOI:** 10.3390/cells14242007

**Published:** 2025-12-16

**Authors:** Aishat O. Ameen, Maja B. Rindshøj, Katarina Stoklund Dittlau, Karin Borges, Kristine K. Freude, Blanca I. Aldana

**Affiliations:** 1 Department of Drug Design and Pharmacology, Faculty of Health and Medical Sciences, University of Copenhagen, DK-2200 Copenhagen, Denmark; 2 Department of Veterinary and Animal Sciences, Faculty of Health and Medical Sciences, University of Copenhagen, DK-1870 Copenhagen, Denmark; 3 School of Biomedical Sciences, Faculty of Medicine, The University of Queensland, Brisbane 4072, Australia

**Keywords:** Alzheimer’s disease, brain energy metabolism, medium-chain fatty acids, decanoic acid, mitochondria, 5xFAD mouse, hiPSC-derived astrocytes

## Abstract

**Highlights:**

**What are the main findings?**

- Medium-chain fatty acid decanoic acid (C10) is efficiently metabolized in both wild-type and 5xFAD mouse brain slices, particularly in astrocytes, supporting mitochondrial function.
- Astrocytic acetate metabolism is impaired in 5xFAD mice and familial AD astrocytes exhibit genotype-dependent metabolic responses to C10, with partial alterations in oxidative glucose metabolism in APP and PSEN1 variants.

**What are the implications of the main findings?**

- Altered astrocytic metabolism might occur before glucose hypometabolism in the 5xFAD mouse model.
- C10 supplementation may provide an auxiliary fuel source capable of supporting brain energy metabolism in Alzheimer’s disease, in part by promoting oxidative metabolism in astrocytes.

**Abstract:**

Alzheimer’s disease (AD) is increasingly recognized as a disorder of cerebral energy metabolism, where impaired glucose utilization contributes to disease pathology. Medium-chain fatty acids (MCFAs), such as decanoic acid (C10), have emerged as promising metabolic substrates due to their ability to bypass glycolytic deficits and support mitochondrial function. In this study, we investigated the metabolic impact of C10 in the 5xFAD mouse model of AD and in human induced pluripotent stem cell (hiPSC)-derived astrocytes carrying familial AD mutations. Utilizing stable ^13^C-labeled metabolic tracers, we demonstrated that while [U-^13^C]glucose metabolism was largely preserved in cortical slices of 6-month-old 5xFAD female mice, [1,2-^13^C]acetate uptake was significantly reduced, suggesting impaired astrocytic metabolism. [U-^13^C]C10 was efficiently metabolized in both WT and 5xFAD brain slices, particularly in astrocytes, as indicated by high labeling of glutamine and citrate. Furthermore, C10 competitively inhibited glucose and acetate metabolism, suggesting its potential as an auxiliary energy substrate. In hiPSC-derived astrocytes, AD-specific metabolic responses to C10 varied by mutation, with only partial alterations in oxidative glucose metabolism observed in APP and PSEN1 variants, highlighting genotype-dependent metabolic alterations. While AD-related mutations in the hiPSC models did not lead to robust deficits, the in vivo environment in the 5xFAD model is associated with measurable metabolic changes in astrocytes. These findings underscore astrocytic metabolic dysfunction in AD and suggest that C10 supplementation may restore brain energy by supporting astrocytic oxidative metabolism.

## 1. Introduction

Alzheimer’s disease (AD) is a neurodegenerative disorder characterized by a loss of memory, cognitive decline, and behavioral changes, which is largely driven by metabolic dysfunction, including impaired cerebral glucose metabolism [1,2]. Although the pathogenesis of AD involves multiple factors, including the accumulation of amyloid-beta plaques, neurofibrillary tangles, and neuroinflammation, emerging research increasingly positions AD as a metabolic disorder of the brain, where energy failure significantly contributes to disease progression [3,4,5]. This view has supported a focus on metabolic interventions to tackle AD pathology, especially the use of alternative energy substrates such as medium-chain fatty acids (MCFAs), including decanoic acid (C10) and octanoic acid (C8) [6].

MCFAs are unique due to their ability to bypass complex digestion pathways and directly enter the liver and brain, where they may act as signaling molecules or undergo oxidation to produce ketone bodies. These MCFAs and ketone bodies, including β-hydroxybutyrate, may serve as alternative energy sources, especially when brain glucose metabolism is compromised, a common trait in AD [7]. Many studies have shown that diets enriched with medium-chain triglycerides (MCTs) containing C8 and C10 can improve cognitive function and brain energy metabolism in individuals with mild cognitive impairment (MCI) and AD [8,9,10].

The metabolism of MCFAs in the brain primarily occurs in astrocytes [11], which play a crucial role in supporting neuronal function. Metabolism of C8 and C10 in astrocytes supports glutamine synthesis, which is then used by neurons to synthesize the neurotransmitter GABA [12,13]. This metabolic coupling between astrocytes and neurons is essential for maintaining amino acid and neurotransmitter balance as well as overall brain health [14]. Additionally, MCFAs have been shown to reduce oxidative stress and inflammation, further contributing to their neuroprotective effects [15,16,17].

Among the MCFAs, C10 has gained attention for its distinct metabolic and potential neuroprotective effects [18,19]. C10 influences not only mitochondrial β-oxidation but also cytosolic pathways, stimulating fatty acid synthesis and potentially enhancing neuronal metabolism. The role of C10 in supporting astrocyte metabolism and fatty acid synthesis may also provide neuroprotective effects. Additionally, we recently showed that C10 rescues differences in AMPA-mediated calcium rises in hippocampal astrocytes and neurons in the 5xFAD mouse model of AD pathology [20].

Despite these promising findings, the exact mechanisms by which MCFAs, particularly C10, exert their beneficial effects in AD remain to be fully elucidated. Here, we investigated brain energy metabolism in the 5xFAD mouse model using uniformly ^13^C-labeled glucose ([U-^13^C]glucose), acetate ([1,2-^13^C]acetate), and C10 ([U-^13^C]C10) to probe neuronal and astrocytic metabolic activity in cerebral slices. We also measured the effect of C10 on [U-^13^C]glucose metabolism in human induced pluripotent stem cell (hiPSC)-derived astrocytes. We demonstrated that astrocytic metabolism was primarily impaired in the cerebral cortex of the 5xFAD model and that C10 could act as an auxiliary brain energy substrate, replacing glucose to some extent. The hiPSC-derived AD astrocytes displayed distinct metabolic alterations in oxidative glucose metabolism upon C10 supplementation; however, these responses varied across AD genotypes, underscoring the complexity of astrocyte metabolic profiles in AD.

## 2. Materials and Methods

### 2.1. Materials

The stable ^13^C isotopes [1,2-^13^C]acetate (CLM-440-SP, sodium salt, 99%), [U-^13^C]decanoic acid ([U-^13^C]C10, CLM-9950-PK, 98%), and [U-^13^C]glucose (CLM-1396-5, 99%) were all obtained from Cambridge Isotope Laboratories (Tewksbury, MA, USA). Acetate (S5636, sodium salt 99%), decanoic acid (^12^C10, C1875), and D-glucose (16301) were purchased from Sigma Aldrich (St. Louis, MI, USA). All other chemicals used were of the purest grade available from commercial sources.

### 2.2. Animals

Female transgenic 5xFAD mice (TG(APPSwFlLon,PSEN1*M146L*L286V)6799Vas, Jax strain: 034840) and wild-type (WT) mice (Jax strain: 100012) were purchased from Jackson Laboratories (Bar Harbor, ME, USA). A colony was bred and maintained at the Department of Drug Design and Pharmacology, University of Copenhagen. The 5xFAD mice express five familial AD mutations in the amyloid precursor protein (APP) and presenillin1 (PSEN1) genes under the neuron-specific Thy1 promoter, leading to rapid brain amyloid deposition [21]. Genotyping of transgenic and WT mice was performed on ear clippings using a standard PCR-based method (JAX protocol 23370), as previously described [13]. For brain slice experiments, female transgenic 5xFAD and WT mice were used at 6 months old, which correlates with increased β-amyloid 42 deposition, cortical and hippocampal plaques, gliosis, and cognitive decline [22]. A total of 12 mice were used in this study, comprising 6 WT and 6 transgenic 5xFAD. The mice were group-housed in individually ventilated cages. All the mice were bred and maintained in a specific pathogen-free, temperature and humidity-controlled environment with a 12 h light/dark cycle. They were given free access to water and chow. The experiments using animals have been reported in compliance with the ARRIVE guidelines.

### 2.3. Brain Slice Incubations

Acute brain slice incubations were performed following established procedures [23]. Mice were euthanized by cervical dislocation and decapitation, after which the brain was rapidly removed and placed in ice-cold, oxygenated artificial cerebrospinal fluid (ACSF; in mM: 128 NaCl, 25 NaHCO_3_, 10 D-glucose, 3 KCl, 2 CaCl_2_, 1.2 MgSO_4_, and 0.4 KH_2_PO_4_; pH 7.4). Cerebral cortices and hippocampi were dissected and sectioned into 350-μm slices using a McIlwain tissue chopper (The Vibratome Company, St. Louis, MI, USA) [13]. Slices were separated under a microscope, and for each incubation condition, two cortical and five hippocampal slices were transferred to custom-made chambers containing 10 mL ACSF at 37 °C, continuously gassed with 95% O_2_/5% CO_2_. Slices were allowed to recover for 60 min [23] before exposure to ACSF supplemented with stable ^13^C-labeled compounds—5 mM [1,2-^13^C]acetate, 5 mM [U-^13^C]glucose, or 200 μM [U-^13^C]C10 (acetate and C10 were supplemented with 5 mM unlabeled D-glucose)—and were then incubated for an additional 60 min. For competition experiments, labelled substrates were co-incubated with their corresponding unlabeled competitors (i.e., [1,2-^13^C]acetate in the presence of unlabeled ^12^C10, [U-^13^C]glucose in the presence of ^12^C-acetate, and [U-^13^C]C10 in the presence of ^12^C-acetate or ^12^C-glucose for 60 min. Incubations were terminated by transferring slices into ice-cold 70% ethanol, followed by sonication and centrifugation (4000× *g*, 20 min). Supernatants were collected, lyophilized, and prepared for gas chromatography–mass spectrometry (GC–MS) analysis.

### 2.4. Cell Lines

AD astrocytes were derived from healthy hiPSCs, in which APP or PSEN1 mutations were introduced via CRISPR-Cas9; all lines were previously characterized [24,25]. The APP Swedish hiPSC line carries a heterozygous KM670/671NL double mutation (BIONi010-C-38, Cell line repository hPSCreg^®^), the APP London line harbors a heterozygous V717I mutation (BIONi010-C-37, Cell line repository hPSCreg^®^), and the PSEN1 line contains a heterozygous E280A mutation (BIONi010-C-30, Cell line repository hPSCreg^®^). The parental hiPSC line BIONi010-C (also known as K3P53, Cell line repository hPSCreg^®^), in which all above mentioned mutations were introduced, was used as the wild-type (WT) control. All cell lines carry the APOE3/4 genotype. hiPSC lines were plated on Geltrex (Thermo Fisher Scientific, Waltham, MA, USA) and cultured in Essential 8 Flex medium (Thermo Fisher Scientific, Waltham, MA, USA). Medium was changed every 2 days until the cells reached 70–90% confluence.

### 2.5. Neural Progenitor Cells Generation and Cell Differentiation

Confluent iPSCs were lifted from Geltrex-coated plates using collagenase type IV (Thermo Fisher Scientific, Waltham, MA, USA) and grown in a 3D culture [26,27]. Neural induction and differentiation were achieved through dual inhibition of SMAD signaling using LDN193189 (Stemgent, Beltsville, MD, USA) and SB431542 (Tocris Bioscience, Bristol, UK) to inhibit the BMP and TGFβ pathway. After 7 days of neuronal induction, embryoid bodies (EBs) were plated on Geltrex-coated plates and supplemented with human epidermal growth factor (EGF) (ProSpec, Ness-Ziona, Israel) and murine fibroblast-basic (FGF) (Peprotech, Cranbury, NJ, USA). Neural rosettes were picked at day 10 for passaging and neural progenitor (NPC) expansion.

### 2.6. Astrocytic Differentiation

The astrocytes were differentiated according to a modified protocol by Shaltouki et al. 2013 [28], as previously described [26]. From day 16 of neural expansion, astrocyte differentiation media (ADM) was added to 90–100% confluent NPCs. ADM contains FGF, human insulin growth factor IGF-1 (IGF) (Peprotech, Cranbury, NJ, USA), Human Activin A recombinant protein (Thermo Fisher Scientific, Waltham, MA, USA), and human Heregulin β-1 (Peprotech, Cranbury, NJ, USA) to promote astrocyte progenitor production. The media was changed every second day until day 25, when gliogenesis was expected to occur, and after which an astrocyte maturation media (AMM) was added. AMM contains growth factors (IGF, Activin A, and Heregulin) and was supplemented with non-essential amino acids, L-Glutamine, sodium pyruvate, and fetal bovine serum (FBS), all purchased from Thermo Fisher Scientific, and L-Ascorbic acid (Sigma-Aldrich, A8960, St. Louis, MI, USA). The astrocytes were matured for 4 weeks prior to conducting metabolic assays. During the maturation period, the astrocytes were passaged biweekly using Accutase. Immunocytochemical characterization of hiPSC-derived astrocytes (Supplementary Methods and Figure S1) was performed as previously described [29].

### 2.7. HiPSC Derived Astrocyte Incubations

hiPSC-derived astrocytes were plated at 30,000 cells/cm^2^ in 6-well plates 48 h before conducting metabolic assays. Cells were rinsed with pre-warmed (37 °C) phosphate-buffered saline (PBS) and incubated for 90 min at 37 °C and 5% CO_2_ in DMEM supplemented with ^13^C-labeled substrates. Astrocytes received either 2.5 mM [U-^13^C]glucose alone or [U-^13^C]glucose together with 200 µM unlabeled C10, corresponding to reported cerebral concentrations following MCT-enriched diets in mice [30,31]. After incubation, the medium was collected, and metabolism was stopped by washing cells with ice-cold PBS. Cells were then lysed and extracted in 70% ethanol, followed by centrifugation at 4 °C (4000× *g*, 20 min). The resulting supernatants containing soluble metabolites were lyophilized and stored at −20 °C until GC–MS analysis.

### 2.8. Metabolic Mapping Using Gas Chromatography Coupled to Mass Spectrometry (GC–MS)

Metabolic ^13^C-enrichment in TCA cycle intermediates was determined GC–MS following established procedures [32]. Lyophilized extracts from brain slices or hiPSC-derived astrocytes were reconstituted in water, acidified, and subjected to two sequential ethanol extractions. Metabolites were further purified using 96% ethanol followed by benzene to obtain the organic phase. The dried extracts were then derivatized using N-tert-butyldimethylsilyl-N-methyltrifluoroacetamide (Sigma Aldrich, St. Louis, MI, USA) prior to GC–MS analysis. The samples were analyzed by GC (Agilent Technologies 7820A chromatograph, J&W GC column HP-5MS, parts no. 19091S-433, Santa Clara, CA, USA) linked to MS (Agilent Technologies 5977E mass spectrometer, Santa Clara, CA, USA). The ^13^C-enrichment was corrected for the natural abundance of ^13^C by analyzing standards of the unlabeled metabolites of interest. Data are expressed as the percentage of labeling in isotopologues of the form M + X, where M represents the molecular mass of the unlabeled molecule, and X denotes the number of ^13^C atoms incorporated. In this study, we focused on M + 3 and M + 2 isotopologues, reflecting metabolites generated during direct metabolism or a first turn of the TCA cycle.

### 2.9. Statistical Analysis

Data are presented as means ± standard deviation (SD), with individual data points shown. A total of 5–6 mice (*n* = 5–6) were used for the brain slice incubations. Each individual data point represents a biological replicate (obtained from an individual animal). 5–6 technical replicates (*n* = 5–6) from 3 independent differentiations were used for the hiPSC-derived astrocyte incubation experiments. Statistical analyses were performed using Graphpad v.10.2.2. An outlier removal (ROUT) test was applied to identify any significant outliers. Outliers were removed from the dataset according to a 1% Q-value. Data were first tested for normality using the Shapiro–Wilk test (*α*  =  0.05). Unpaired data were compared using either a two-tailed Welch’s *t*-test or Mann–Whitney test. The significance level was set at *p*  <  0.05 and is indicated as *, *p*  <  0.05, **, *p* < 0.01, ***, *p* < 0.001 ****, *p* < 0.0001. For details on the statistical analyses, see Supplementary Table S1–S15. Although brain slices from WT and 5xFAD mice were exposed to the same ^13^C-labeled substrates, randomization was applied to minimize potential bias in sample handling and processing. Mice were randomly selected for each experimental run by the experimenters. Similarly, for hiPSC-derived astrocyte experiments, wells were randomly assigned to treatment conditions. The study did not apply stratification or blocking, as all animals and cell cultures were treated under uniform conditions using standardized protocols. Cage location was not controlled, but uniform housing and standardized protocols were maintained to reduce confounding effects. Blinding was implemented at multiple stages of the study. Investigators conducting the GC–MS analysis and statistical evaluation were blinded to the group allocations. Sample tubes were coded, ensuring that the identity of the treatment groups remained concealed until after data processing was complete. Blinding was not applied during animal allocation or experimental procedures due to logistical constraints.

## 3. Results

### 3.1. Acetate Metabolism Is Impaired in Cerebral Cortical Slices of 5xFAD Mice While Glucose Metabolism Is Maintained

**Figure 1:**
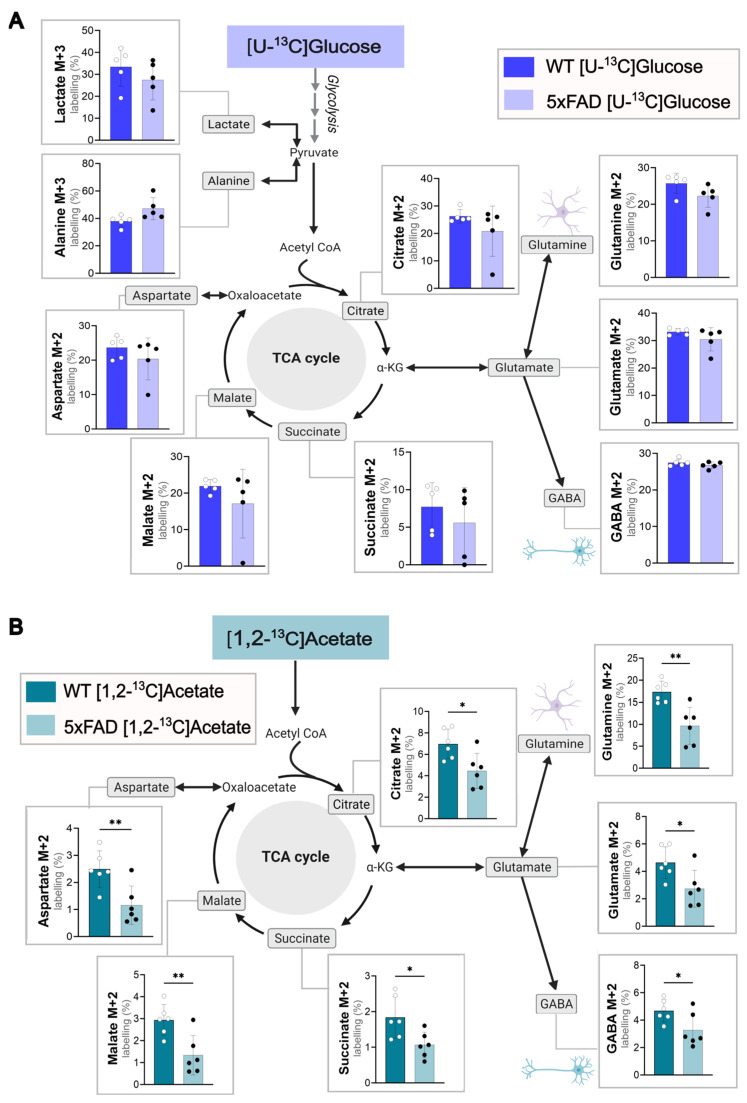
Glucose and acetate metabolism in WT and 5xFAD mouse cerebral cortex slices. Metabolism of (**A**) [U-^13^C]glucose or (**B**) [1,2-^13^C]acetate in acutely isolated cerebral cortical slices of 6-month-old WT and 5xFAD mice. Labeling in glycolytic products (M + 3) and first turn (M + 2) TCA cycle intermediates shown. Slices were incubated with medium containing 5 mM [U-^13^C]glucose or 5 mM [1,2-^13^C]acetate (supplemented with 5 mM D-glucose), and cell extracts were analyzed via GC-MS. Mean  ±  SD, *n* = 5–6 from individual animals, Welch’s *t*-test or Mann–Whitney test, * *p* < 0.05, ** *p* < 0.01.

To validate changes in cellular energy metabolism in 5xFAD mice compared to WT, acutely isolated cerebral cortical (Figure 1) and hippocampal slices (Figure S2) were incubated in the presence of ^13^C-labeled energy substrates to functionally explore metabolism. Glucose is the main energy substrate of the brain, and the metabolism of [U-^13^C]glucose provides an overview of metabolic function in the brain slices [23]. In both the WT and 5xFAD brain slices, [U-^13^C]glucose was incorporated via glycolysis, giving rise to labeling in lactate (M + 3) and alanine (M + 3) as well as labelled pyruvate, which enters the TCA cycle as acetyl co-enzyme A (M + 2). No difference was observed between 6-month-old WT and 5xFAD mice in the metabolism of [U-^13^C]glucose in cortical brain slices (Figure 1A). However, reductions in the ^13^C labeling of alanine, malate, and glutamate were observed in 5xFAD hippocampal (Figure S2) brain slices. When the slices were incubated with [1,2-^13^C]acetate, a substrate primarily metabolized in astrocytes [33], a marked decrease in ^13^C enrichment of all TCA cycle intermediates (citrate, succinate, and malate) and derived amino acids (glutamate, glutamine, GABA, and aspartate) was seen in 5xFAD cerebral cortical slices compared to WT (Figure 1B). The largest reduction in ^13^C-enrichment (%) was observed in malate (*p* = 0.0064) and the derived amino acids aspartate (*p* = 0.0075) and glutamine (*p* = 0.0031). No alterations in hippocampal [1,2-^13^C]acetate metabolism were detected between WT and 5xFAD mice (Figure S2). These findings indicate a region-specific hampering of acetate metabolism in the cortical astrocytes of 5xFAD mice and glucose metabolism in their hippocampi.

### 3.2. Decanoic Acid Can Be Used as a Brain Energy Substrate and Can Compete with Glucose and Acetate to Support Brain Energy Metabolism

**Figure 2:**
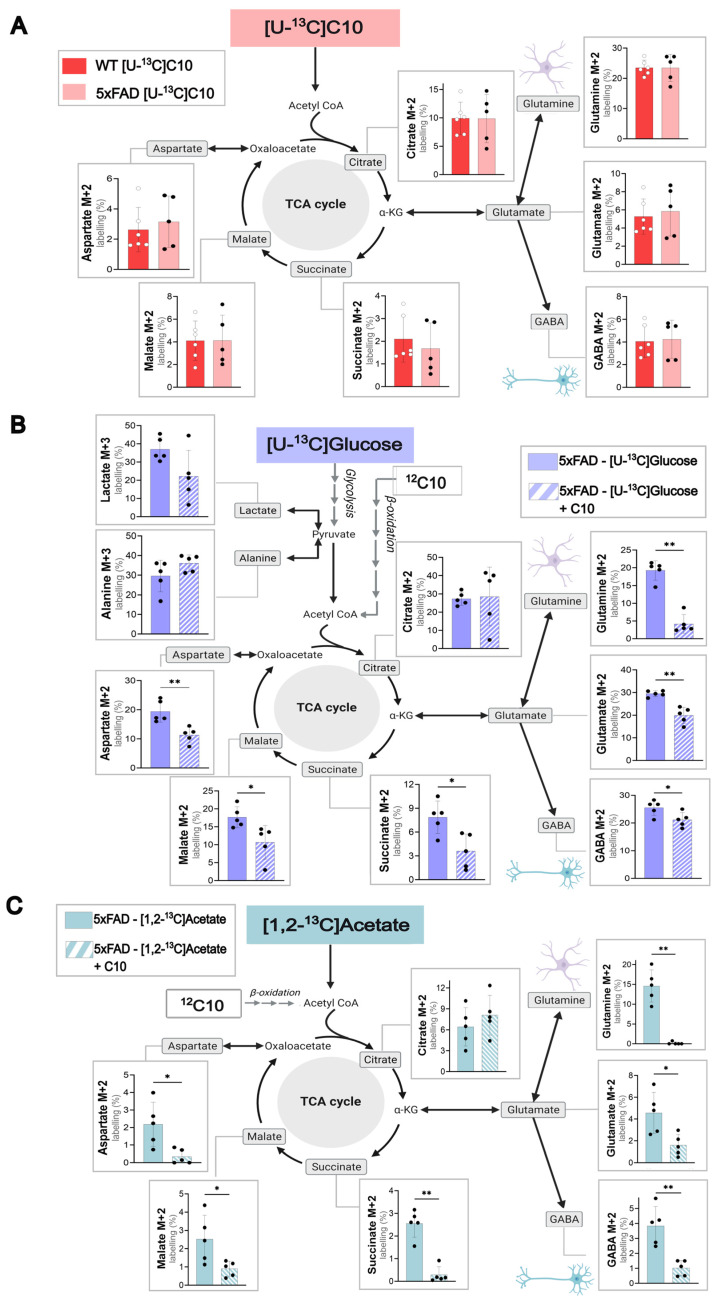
C10 as a metabolic substrate in WT and 5xFAD mouse cerebral cortex slices. (**A**) metabolism of 0.2 mM [U-^13^C]C10 in acutely isolated cerebral cortical slices of WT and 5xFAD mice. (**B**) Metabolic competition assay between [U-^13^C]glucose and unlabeled ^12^C10 in cerebral cortical slices of 5xFAD mice. (**C**) Metabolic competition assay between [1,2-^13^C]acetate and unlabeled ^12^C10 in cerebral cortical slices of 5xFAD mice. Glycolytic products (M + 3) and first turn (M + 2) TCA cycle intermediates are shown. Concentrations: 5 mM glucose, 5 mM acetate, and 0.2 mM C10. Unless incubated with [U-^13^C]glucose, all incubations were supplemented with 5 mM D-glucose. Mean  ±  SD, *n* = 5–6 from individual animals, Welch’s *t*-test or Mann–Whitney test, * *p* < 0.05, ** *p* < 0.01.

C10 can act as an auxiliary brain energy substrate, as demonstrated in previous experiments [12]. Yet the cellular energy metabolism of C10 in the 5xFAD mouse model is still mostly unexplored. To study the differences in C10 metabolism in a pathological setting, acutely isolated cerebral cortical (Figure 2A) and hippocampal slices (Figure S3) from 5xFAD and WT mice were incubated with labeled [U-^13^C]C10. [U-^13^C]C10 enters cellular metabolism as ^13^C_2_-acetyl CoA units, resulting in ^13^C-enrichment in TCA cycle intermediates and derived amino acids. Incorporation of ^13^C due to C10 metabolism was seen in all TCA cycle intermediates and derived amino acids for both wild-type and 5xFAD mice (Figure 2A), verifying the ability of C10 to be used as an energy substrate for metabolism in both mouse genotypes. Interestingly, the highest first turn labeling (%) following metabolism of [U-^13^C]C10 was seen in the amino acid glutamine M + 2 (WT and 5xFAD; 23.53 ± 1.02% and 23.48 ± 2.00%). This indicates that C10 is metabolized to a higher degree in astrocytes than neurons, as the enzyme GS, which converts glutamate to glutamine, is selectively expressed in astrocytes [34]. High ^13^C-enrichment (%) was also seen in the TCA cycle intermediate citrate M + 2, which is also suggested to be an indicator for astrocyte metabolism [23,35]. These results support the view that C10 is mainly metabolized in astrocytes. [12]

To study the direct effect of C10 on glucose metabolism, brain slices from 5xFAD and WT mice were incubated with labeled [U-^13^C]glucose and unlabeled ^12^C10 in a competition assay. As the ^13^C10 enrichment levels in the tested metabolites (Figure 2A) were similar in WT and 5xFAD mice, the results below will focus on the 5xFAD mouse model (WT values in Figure S3B). Cortical slices incubated with [U-^13^C]glucose in the presence of unlabeled ^12^C10 showed a reduction of ^13^C-enrichment in the TCA cycle intermediates (succinate and malate) and the amino acids (glutamine, glutamate, GABA, and aspartate). The results indicate that C10 can compete with glucose as an energy substrate in the brain. By competing with glucose, the ^12^C carbons from C10 dilute the ^13^C-enrichment in the TCA cycle intermediates and amino acids from [U-^13^C]glucose. The significant decrease in ^13^C-enrichment was pronounced in the TCA cycle-derived amino acid glutamine M + 2 (*p* = <0.01). These findings correlated with the data in Figure 2A of the preferential metabolism of C10 by astrocytes. The same tendency was observed in WT and 5xFAD hippocampal slices metabolizing glucose (Figure S4).

As C10 can compete with glucose as a substrate for energy metabolism, we wanted to investigate whether C10 could influence acetate metabolism. Cortical brain slices of WT (Figure S3) and 5xFAD (Figure 2C) mice were incubated with labeled [1,2-^13^C]acetate and unlabeled ^12^C10. The labeling patterns for WT and 5xFAD mice were similar, so the following results focus on the 5xFAD mouse model. Figure 2C illustrates an overall decrease in ^13^C-enrichment derived from [1,2-^13^C]acetate when unlabeled C10 is present in the incubation medium. The most significant reduction in labeling (%) was seen in the TCA cycle intermediate succinate M + 2 (*p* = < 0.01) and the amino acid glutamine M + 2 (*p* = < 0.01). The same experiment was completed with hippocampal slices of WT and 5xFAD mice, which showed the same tendencies (Figure S4). The results strongly suggest that C10 can compete with acetate as an astrocyte energy substrate in cortical brain slices.

### 3.3. The Effect of C10 on Glucose Metabolism Is AD Mutation-Dependent in hiPSC-Derived Astrocytes

**Figure 3:**
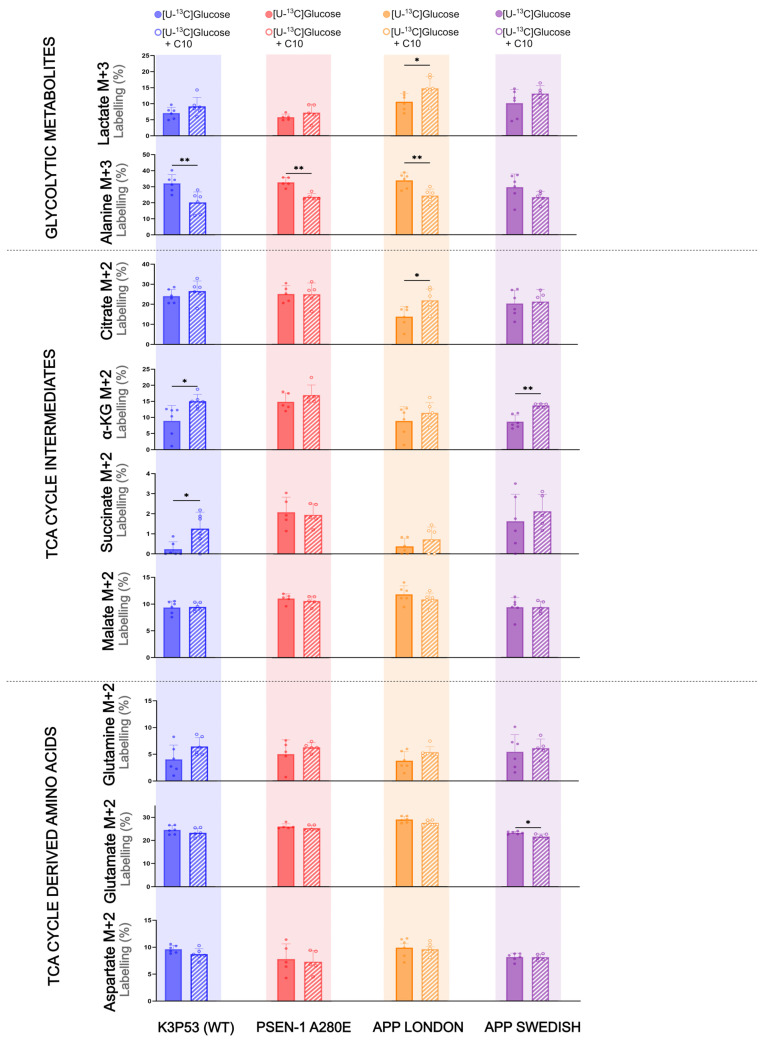
The Influence of C10 on glucose metabolism in hiPSC-derived astrocytes carrying AD mutations. Metabolism of [U-^13^C]glucose in the presence and absence of ^12^C-C10 in hiPSC-derived astrocytes AD with a PSEN-1 or APP mutation, compared to WT. Glycolytic products (M + 3) and first turn (M + 2) TCA cycle intermediates are shown. Concentrations: 2.5 mM [U-^13^C]glucose or 2.5 mM [U-^13^C]glucose with 0.2 mM ^12^C-C10. Mean  +  SD, *n* = 3 independent differentiations; *n* = 5–6 technical replicates, Welch’s *t*-test or Mann–Whitney test, * *p* < 0.05, ** *p* < 0.01.

The incubation experiments in the 5xFAD brain slices showed that C10 could act as an energy substrate as well as affect glucose metabolism. These effects of C10 on cellular brain metabolism are most prominent in astrocytes; thus, we sought to determine whether C10 affects glucose metabolism in hiPSC-derived astrocytes. HiPSC-derived astrocytes with mutations in the amyloid precursor protein (APP) or presenilin-1 (PSEN-1) genes were used as a human cell model for familial forms of AD. HiPSC-derived astrocytes, K3P53 (referred to as WT astrocytes), and astrocytes carrying PSEN-1 A280E, APP London, or APP Swedish mutations (referred to as AD astrocytes) were incubated with [U-^13^C]glucose in the absence and presence of unlabeled ^12^C10 (Figure 3). When incubated with [U-^13^C]glucose in the presence of ^12^C10, WT astrocytes displayed increased ^13^C enrichment of α-ketoglutarate (*p* = 0.024) and succinate (*p* = 0.043) compared to enrichment from [U-^13^C]glucose alone. ^13^C enrichment for the remaining TCA cycle intermediates in the WT astrocytes was unaltered in response to ^12^C10. APP London astrocytes showed higher labeling in lactate (*p* = 0.049) and citrate ( *p*= 0.024) from [U-^13^C]glucose in the presence of ^12^C10 than from [U-^13^C]glucose. APP Swedish astrocytes incubated with [U-^13^C]glucose in the presence of ^12^C10 only resulted in increased labeling for α-ketoglutarate (*p* = 0.004). Interestingly, the presence of ^12^C10 slightly lowered glutamate labeling (*p* = 0.025) derived from [U-^13^C]glucose that was found in APP Swedish astrocytes. ^13^C enrichment for TCA cycle intermediates and TCA cycle-derived amino acids remained unchanged in PSEN-1 A280 astrocytes incubated in [U-^13^C]glucose media with or without ^12^C10. However, PSEN-1 A280E, along with WT and APP London astrocytes, showed lower labeling in alanine (*p* = 0.0079, *p* = 0.0067, *p* = 0.0039, respectively) when incubated with [U-^13^C]glucose in the presence of ^12^C10. Together, these results suggest that oxidative metabolism of glucose in these AD astrocytes remains mostly unchanged upon addition of C10, particularly in the PSEN-1 mutant.

## 4. Discussion

In this study, we show that while glucose metabolism was largely maintained in both WT and 5xFAD cerebral cortical slices, acetate metabolism in 5xFAD cortices was lower compared to WT. Additionally, the MCFA C10 could act as an auxiliary brain energy substrate in both WT and 5xFAD. In hiPSC-derived AD astrocytes, there were distinct metabolic alterations in oxidative glucose metabolism upon C10 supplementation, though these responses varied across AD genotypes, emphasizing the complexity of astrocyte metabolic adaptations in AD.

### 4.1. Regional Changes in 5xFAD Brain Energy Metabolism

In our study, we did not observe a change in cortical [U-^13^C]glucose metabolism between WT and 5xFAD mice at 6 months of age (Figure 1A). There were, however, reductions in [U-^13^C] glucose-derived ^13^C- enrichment of TCA cycle intermediates in 5xFAD hippocampi, relative to WT (Figure S2). In a study specifically analyzing glucose uptake in the 5xFAD mouse model, despite increased Aβ burden in the brains at 2 and 5 months, it was only at 13 months that significant reductions to glucose metabolism could be seen in the whole brain and specific regions [36]. Another study looking at [^18^F]FDG utilization in 5xFAD mice found no change in uptake at 4 months; however, there was increased hippocampal [^18^F]FDG uptake at 8 and 12 months in 5xFAD compared to WT [37]. Hypermetabolic states have been reported in different brain regions, particularly in the early stages of human AD, furthering the need for more longitudinal studies into the glucose metabolism in different AD mouse models and how these compare to the human disease [38,39]. One of the reasons postulated for relatively preserved glucose uptake is microglial reconfiguration in the hippocampus, driving glucose uptake [37,40]. Also, because [^18^F]FDG uptake patterns on PET assess the hippocampus as a whole, the influence of different cell types cannot be distinguished. The use of labeled stable isotopes to map metabolism offers greater insight into oxidative metabolism in AD. In a previous study, we observed a hypometabolism of [U-^13^C]glucose in hippocampal slices and, to a lesser extent, cortical slices of 8-month-old 5xFAD mice [13] which agrees with what we see in the AD hippocampi of this study. Despite the 5xFAD model showing significant Aβ accumulation and gliosis from the age of 2 months [21] it is possible that with increased age, greater neurodegeneration exacerbates hypometabolism. Human studies demonstrating early changes to glucose metabolism also rely on relatively large sample sizes [41] A larger sample size in our study may have allowed detection of impaired glucose metabolism in our young 5xFAD mice.

The uptake of [1,2-^13^C]acetate, a substrate primarily metabolized in astrocytes, was used to investigate cell-specific metabolism [42]. In our study, we showed significantly lower labeling percentages in all metabolites of the TCA cycle and amino acids derived from [1,2-^13^C]acetate metabolism in 5xFAD cortices compared to WT (Figure 1B). This finding strongly indicates hampered astrocytic metabolism in the 6-month-old 5xFAD mice. This correlates with the astrogliosis found to occur in the 5xFAD model from as early as 2 months old [21] and previous studies where we have seen low ^13^C-enrichment of glutamine from [U-^13^C]glucose metabolism in 5xFAD mice [13,43]. Astrogliosis can occur as a response to Aβ pathology, as astrocytes near Aβ plaques become hypertrophic and proliferative [44], later leading to synaptic dysfunction and altered neurotransmission in AD [45,46,47]. Reactive astrocytes can become dysfunctional, with reduced ability to support neurons through metabolic coupling and neurotransmitter recycling [48]. From the findings in this study, it could be suggested that altered astrocytic metabolism might occur before glucose hypometabolism in the 5xFAD mouse model.

### 4.2. C10 as a Brain Substrate in AD and Its Effect on Glucose and Acetate Metabolism

Several studies have highlighted the benefits of MCFAs in neurological disorders, in particular MCFAs as brain energy substrates [16,49,50]. Through dynamic isotope labeling, we previously demonstrated that C10 is preferred over octanoic acid (C8) as a metabolic substrate in the cerebral cortex of NMRI mice and improved mitochondrial respiration [12]. The direct effect of C10 as a metabolite in an AD mouse model, and specifically the 5xFAD brain model, has remained relatively unexplored, so the next aim of our study was to first verify the extent to which C10 was metabolized under pathophysiological conditions. The use of [U-^13^C]C10 as an energy substrate was confirmed in brain slices of 5xFAD and WT mice (Figure 2A and Figure S3). Furthermore, no differences between WT and 5xFAD mice were observed in the ^13^C-enrichment of the TCA cycle intermediates and derived amino acids obtained from [U-^13^C]C10. This indicates that the two types of mice were equally effective in utilizing the MCFA as a brain energy substrate. These results agree with another study testing tridecanoin (the triglyceride of C10) in an epilepsy mouse model [17]. Tridecanoin was an anticonvulsant and improved mitochondrial function in the disease model. Though direct tracking of labeled MCFAs in the brain was not conducted, levels of C10 were elevated in the mice fed with the dietary supplement, suggesting a sustained ability to take up this substrate [17]. These findings are also in line with research suggesting that MCFAs could act as an alternative substrate during reduced glucose utilization, by bypassing the impaired enzymes associated with glycolysis [50,51,52]. In our study, the ^13^C-enrichment from [U-^13^C]C10 metabolism was highest in the amino acid glutamine with respect to other metabolites. This indicates that C10 is mainly metabolized in astrocytes, as glutamine is synthesized in astrocytes [23,53]. ^13^C-enrichment in the amino acid GABA via [U-^13^C]C10 metabolism was similar to what we have seen from [1,2-^13^C]acetate but much lower than enrichment from [U-^13^C]glucose (Figure 1 and Figure 2), which aligns with C10 being primarily metabolized in astrocytes. GABA is synthesized in GABAergic neurons via the use of astrocyte-derived glutamine, which is converted to glutamate via phosphate-activated glutaminase (PAG) and subsequently to GABA via glutamate decarboxylase (GAD). This astrocyte-derived glutamine is thought to be an essential substrate for neuronal GABA synthesis [54,55,56]; thus, the ^13^C-enrichment in GABA in our study likely comes from the C10-derived glutamine via the glutamate/GABA-glutamine cycle.

Our results also showed that unlabeled ^12^C10 reduced [U-^13^C]glucose metabolism in both WT and 5xFAD mice. This was detected by significantly lower ^13^C-enrichment in almost all metabolites from [U-^13^C]glucose metabolism when ^12^C10 was present in the incubation medium (Figure 2B). The same effect was seen when [1,2-^13^C]acetate was metabolized in the presence of ^12^C10. Though the majority of glucose in the brain is utilized by neurons for oxidative phosphorylation [57], astrocytes also metabolize glucose; hence, some of the competition between [U-^13^C]glucose and ^12^C10 could be attributed to astrocytes. It is also possible that neurons are able to metabolize C10 as demonstrated by the metabolism of C8 in hypothalamic neurons [58], however more research is needed in this area. Metabolic competition between [1,2-^13^C]acetate and ^12^C10 again substantiates the findings pointing to astrocytes as the main compartment for C10 metabolism. Competition between [1,2-^13^C]acetate and ^12^C10 was greater than between [U-^13^C]glucose and ^12^C10, characterized by a greater reduction in [1,2-^13^C]acetate labeling in the presence of C10 (Figure 2C).

Finally, our current metabolic findings may provide a mechanistic context for the functional effects of C10 on AMPA-mediated calcium signaling that we previously reported in hippocampal astrocytes (and neurons) from 5xFAD mice [20]. In that study, C10 exposure restored reduced AMPA-evoked calcium transients in both cell types to wild-type levels, suggesting improved excitatory signaling and cellular responsiveness. The present isotopic enrichment data indicate that C10 is readily oxidized in both wild-type and 5xFAD tissue and can partially compete with glucose and acetate for entry into central metabolic pathways. This efficient oxidative utilization of C10, particularly within astrocytic TCA cycle intermediates such as citrate and glutamine, supports the idea that C10 enhances metabolic flexibility and mitochondrial substrate availability. Such metabolic support may, in turn, facilitate the restoration of calcium handling and receptor function observed previously. Thus, when considered together, our functional and metabolic results suggest that C10 may modulate neuronal and astrocytic signaling roles, at least in part, by maintaining the energetic state of the tissue, enabling more robust receptor-mediated signaling in the AD context. However, further research is needed to determine how C10 affects cell-specific functional outcomes across different populations and pathological stages.

### 4.3. Effect of C10 on Glucose Metabolism in hiPSC Derived Astrocytes Carrying AD Mutations

Findings from the incubation experiments on hiPSC-derived astrocytes did not reveal differences in glucose metabolism between WT and AD mutants (Figure 3). Considering the reduction in acetate labeling in 5xFAD cortical slices compared to WT (Figure 1B). Other studies analyzing the metabolic activity of hiPSC PSEN, APP, or late onset AD (LOAD) mutant astrocytes via Seahorse have demonstrated increased oxidative consumption rates (OCR) and extracellular acidification rates (ECAR) compared to controls [29,59,60]. Coupled with increased ROS, oxidative stress, and reduced lactate production in AD astrocytes, this dysfunction could contribute to AD pathology [59]. While APP and PSEN1 mutations classically drive neuronal Aβ overproduction, accumulating evidence indicate that familial AD mutations can modulate astrocyte biology independently of Aβ secretion [29,61]. Together, this highlights the relevance of further modeling mutation-specific pathological changes in specific cellular populations. Our hiPSC-derived astrocyte model represents an early stage of AD. With extended maturation, these cells may develop additional metabolic dysfunction, as reported in studies using AD astrocytes matured for 7 weeks, and 5–8 months [59,62]. Mitochondrial stress tests also stimulate astrocytes, which could highlight metabolic differences compared to WT. Stimulation of astrocytes with inflammatory microglial factors such as IL-1α and TNFα increases glycolytic activity but not mitochondrial respiration [63]. The influence of C10 on glucose metabolism is less conclusive; our results indicate that while C10 partially modulated oxidative glucose metabolism in WT and certain AD-mutant astrocytes, they generally exhibited minimal metabolic adaptation to C10. Our observations that C10 increased ^13^C enrichment in certain TCA cycle intermediates and related metabolites in AD-mutant astrocytes are intriguing and suggest a more complex metabolic interaction. Rather than simply diluting the ^13^C label, C10 may stimulate glucose metabolism or hinder competing pathways, leading to increased flux of ^13^C from glucose, paradoxically increasing ^13^C enrichment into specific metabolic pools probing astrocytic metabolic compartmentalization. C10 may act as a metabolic modulator, enhancing mitochondrial function or altering substrate preference in a mutation-dependent manner. While hiPSC-derived AD astrocytes show no baseline glucose metabolism differences from WT under unstimulated conditions, metabolic alterations may arise with maturation or stress. C10 modulates glucose metabolism in a mutation-dependent way, highlighting metabolic heterogeneity among AD genotypes and underscoring the need for further investigation.

Astrocytic identity and reactivity are important considerations when interpreting metabolic phenotypes in both brain slices and hiPSC-derived astrocytes. We have previously characterized GFAP expression in the 5xFAD model, demonstrating robust and age-dependent astrogliosis in cortical and hippocampal regions [22], and we have likewise documented stable GFAP and S100β expression across hiPSC-derived astrocyte differentiation batches [29], which we also validated in the current cultures (Supplementary Figure S1). These earlier studies establish both the astrocytic purity of the cultures and the expected reactive profile in the 5xFAD model. As mentioned earlier, reactive astrocytes undergo well-described metabolic reprogramming that can influence substrate handling in AD models. Evidence shows that astrocytic reactivity is associated with increased glycolytic flux and reduced oxidative TCA cycle activity [64]. Reactive states also alter glutamate–glutamine cycling and anaplerosis, often lowering glutamine synthesis and modifying aspartate and citrate labeling patterns [13]. These mechanisms are consistent with the reduced [1,2-^13^C]acetate utilization observed in 5xFAD slices and may reflect gliosis-driven changes in astrocytic acetyl-CoA metabolism. In contrast, hiPSC-derived astrocytes, showing limited spontaneous reactivity, displayed only subtle, mutation-dependent metabolic shifts, suggesting that acute reactivity may exert a stronger influence on substrate handling than cell-autonomous effects of familial AD mutations. This highlights astrocytic reactivity as a potential modulator of MCFA and acetate metabolism.

Although our isotopic approach strongly indicates astrocytic involvement, particularly through glutamine labeling patterns, cortical slice experiments inherently include multiple viable cell types, including neurons and other types of glial cells. Therefore, the observed metabolic alterations in the 5xFAD tissue could also arise from indirect, non-cell-autonomous effects driven by incipient neuronal dysfunction or neuroinflammatory signaling. The reduced [1,2-^13^C]acetate metabolism in 5xFAD slices is consistent with impaired astrocytic function, yet we cannot exclude contributions from altered neuron–astrocyte metabolic coupling or microglial reprogramming. This contrasts with our isolated hiPSC-derived astrocyte data, where AD mutations alone only partially influence oxidative metabolism, suggesting that the in vivo disease microenvironment, including cellular cross-talk, inflammatory cues, and amyloid pathology, may be required to elicit the metabolic phenotype we detect in the 5xFAD model. Future studies employing astrocyte-specific metabolic reporters or cell-type-specific tracing strategies will be critical to dissect the relative contributions of each cell population to the metabolic changes observed.

In conclusion, using 5xFAD mice and human iPSC-derived astrocytes with familial AD mutations, our study found that C10 can compete with glucose and acetate as an energy substrate. Our findings suggest that C10 could act as an auxiliary substrate to help restore brain energy fitness in AD, highlighting its therapeutic potential.

## Data Availability

The original contributions presented in this study are included in the article and Supplementary Material. Further inquiries can be directed to the corresponding author.

## Supplementary Materials

The following supporting information can be downloaded at: https://www.mdpi.com/article/10.3390/cells14242007/s1. Figure S1. Immunocytochemistry labelling of hiPSC-astrocytes; Figure S2. Glucose and acetate metabolism in WT and 5xFAD hippocampal brain slices; Figure S3. C10 as a metabolic substrate in mouse hippocampal and cortical slices; Figure S4. Metabolic competition assay between glucose and C10 in mouse hippocampal slices; Tables S1–S15. Full Statistical Report.

## Abbreviations

The following abbreviations are used in this manuscript:

^13^C	Carbon-13 isotope
ACSF	Artificial cerebrospinal fluid
AD	Alzheimer’s disease
APP	Amyloid precursor protein
APOE	Apolipoprotein E
C10	Decanoic acid
DMEM	Dulbecco’s Modified Eagle Medium
FAD	Familial Alzheimer’s disease
GABA	Gamma-aminobutyric acid
GC–MS	Gas chromatography–mass spectrometry
hiPSC	Human induced pluripotent stem cell
MCI	Mild cognitive impairment
MCFA	Medium-chain fatty acid
MCT	Medium-chain triglyceride
PSEN1	Presenilin 1
SMAD	Small mothers against decapentaplegic
TCA	Tricarboxylic acid cycle
