# Supplementary material for: Metabolic Astrocytic Support with Decanoic Acid Enhances Energy Metabolism in Alzheimer’s Disease Models
Source: Cells. 2025 Dec 16;14(24):2007. doi: 10.3390/cells14242007 (PMC12731942; doi:10.3390/cells14242007)
Supplement: Supplementary file 1 [file cells-14-02007-s001.zip › cells-3989966-supplementary.pdf]

Metabolic Astrocytic Compensation with Decanoic Acid Enhances Energy Metabolism in Alzheimer's Disease Models

Aishat O. Ameen, Maja B. Rindshøj, Katarina Stoklund Dittlau, Karin Borges, Kristine K. Freude and Blanca I. Aldana

Supplementary methods

Immunohistochemistry

Immunocytochemical characterization of hiPSC-derived astrocytes was performed to validate the composition of our cultures. Cells were fixed in 4% PFA for 20 min, permeabilized in PBS with 0.2% Triton X-100 for 20 min, and blocked in 3% BSA for 30 min. Primary antibodies were applied overnight at 4°C, including GFAP (1:500, Agilent Dako, Z0334), S100B (1:1000, Sigma, S2532), SOX9 (1:200, Cell Signaling, 82630S) and vimentin (Thermo Fisher Scientific, 1:500, 18-0052). After PBS-Triton washes, cells were incubated with Alexa Fluor 594 (A21125, A32740) or Alexa Fluor 488 (A11008, A32731) secondary antibodies for 1 h at room temperature, followed by DAPI staining (Thermo Fisher, S33025). Coverslips were mounted using Dako mounting medium (Agilent, S3023). Imaging was performed on a on a Leica DMRB-fluorescence microscope at wavelengths 488 nm and 594 nm 40x magnification. Images were processed Fiji ImageJ version 1.54g

Supplementary data

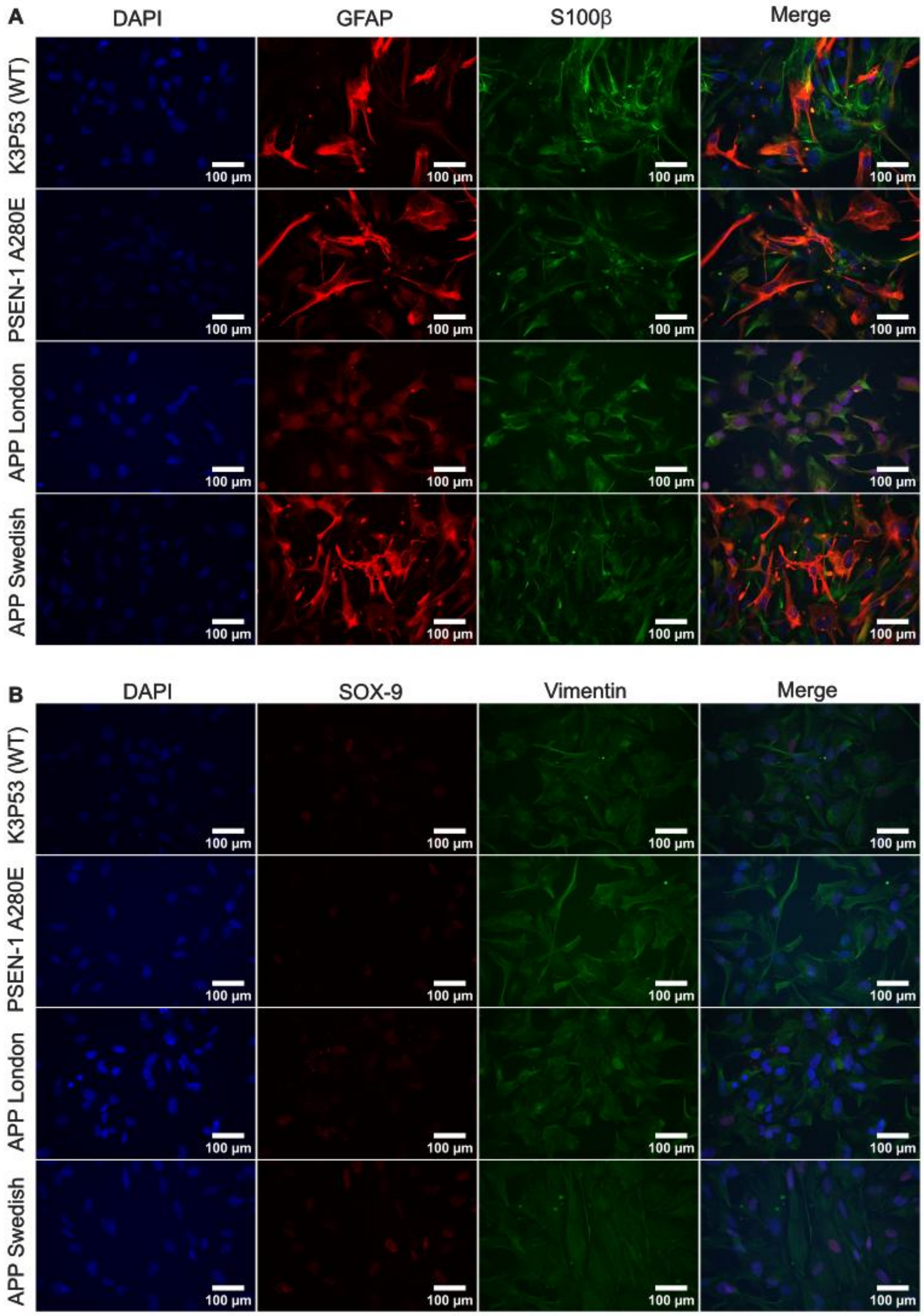

Figure S1. Immunocytochemistry labelling of hiPSC-astrocytes. Representative fluorescence microscopy images of human induced pluripotent stem cell (hiPSC)-derived astrocytes (K3P53 (WT), PSEN-1 A280E, APP London and APP Swedish) after 4 weeks of maturation. Cells were stained with astrocyte-specific markers **(A)** GFAP, S100β, **(B)** SOX-9 and vimentin. Nuclei stained with DAPI. Scale bar = 100 µm.

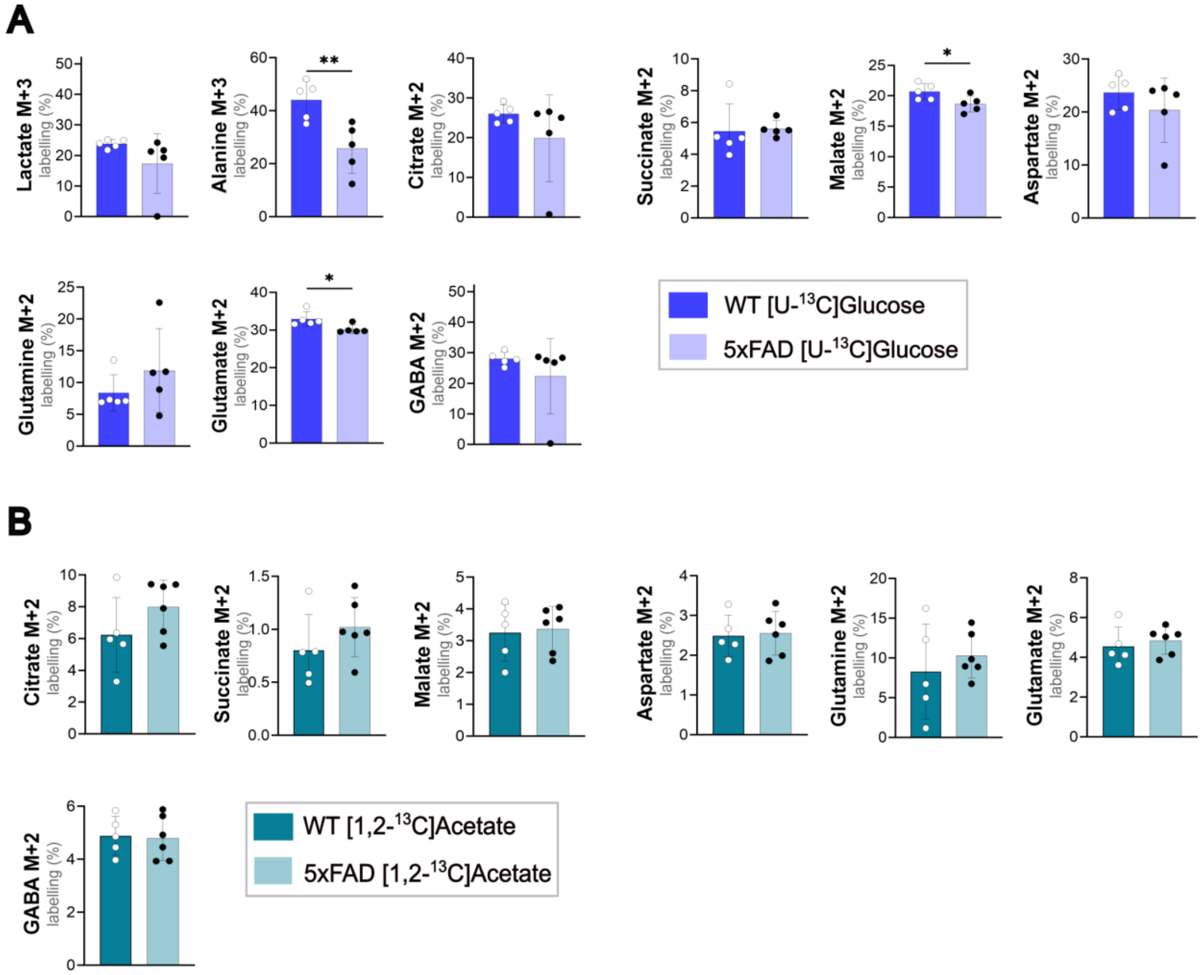

Figure S2. Glucose and acetate metabolism in WT and 5xFAD hippocampal brain slices. (A) metabolism of [U-^{13}C]glucose in acutely isolated cerebral hippocampal slices of 6 month old WT and 5xFAD mice. **(B)** metabolism of [1,2-^{13}C]acetate in acutely isolated cerebral hippocampal slices of 6 month old WT and 5xFAD mice. Glycolytic products (M+3) and first turn (M+2) TCA cycle intermediates shown. Concentrations: 5 mM [U-^{13}C]glucose or 5 mM [1,2-^{13}C]acetate (supplemented with 5 mM D-glucose). Mean ± SD, n = 5-6 from individual animals, Welch's t-test or Mann–Whitney test, *$p < 0.05$, **$p < 0.01$.

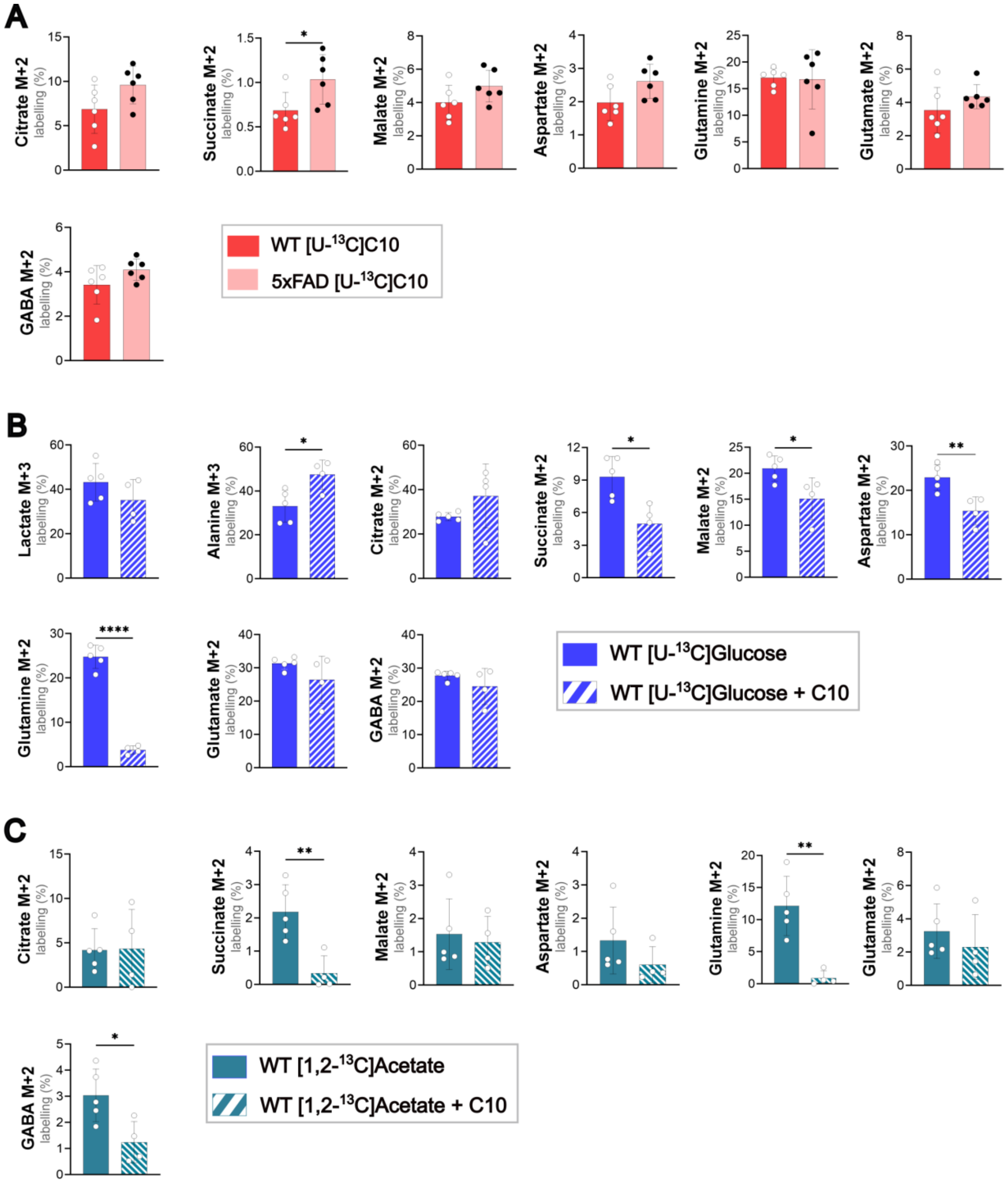

Figure S3. C10 as a metabolic substrate in mouse hippocampal and cortical slices. (A) metabolism of 0.2 mM [U-^{13}C]C10 in acutely isolated hippocampal slices of WT and 5xFAD mice. **(B)** Metabolic competition assay between [U-^{13}C]glucose and unlabelled ^{12}C10 in cerebral cortical slices of WT mice. **(C)** Metabolic competition assay between [1,2-^{13}C]acetate and unlabelled ^{12}C10 in cerebral cortical slices of WT mice. Glycolytic products (M+3) and first turn (M+2) TCA cycle intermediates shown. Concentrations: 5 mM glucose, 5 mM acetate and 0.2 mM C10. Unless incubated with [U-^{13}C]glucose, all incubations were supplemented with 5 mM D-glucose. Mean ± SD, n = 4-5 from individual animals, Welch's t-test or Mann–Whitney test, *$p < 0.05$, **$p < 0.01$, ****$p < 0.0001$.

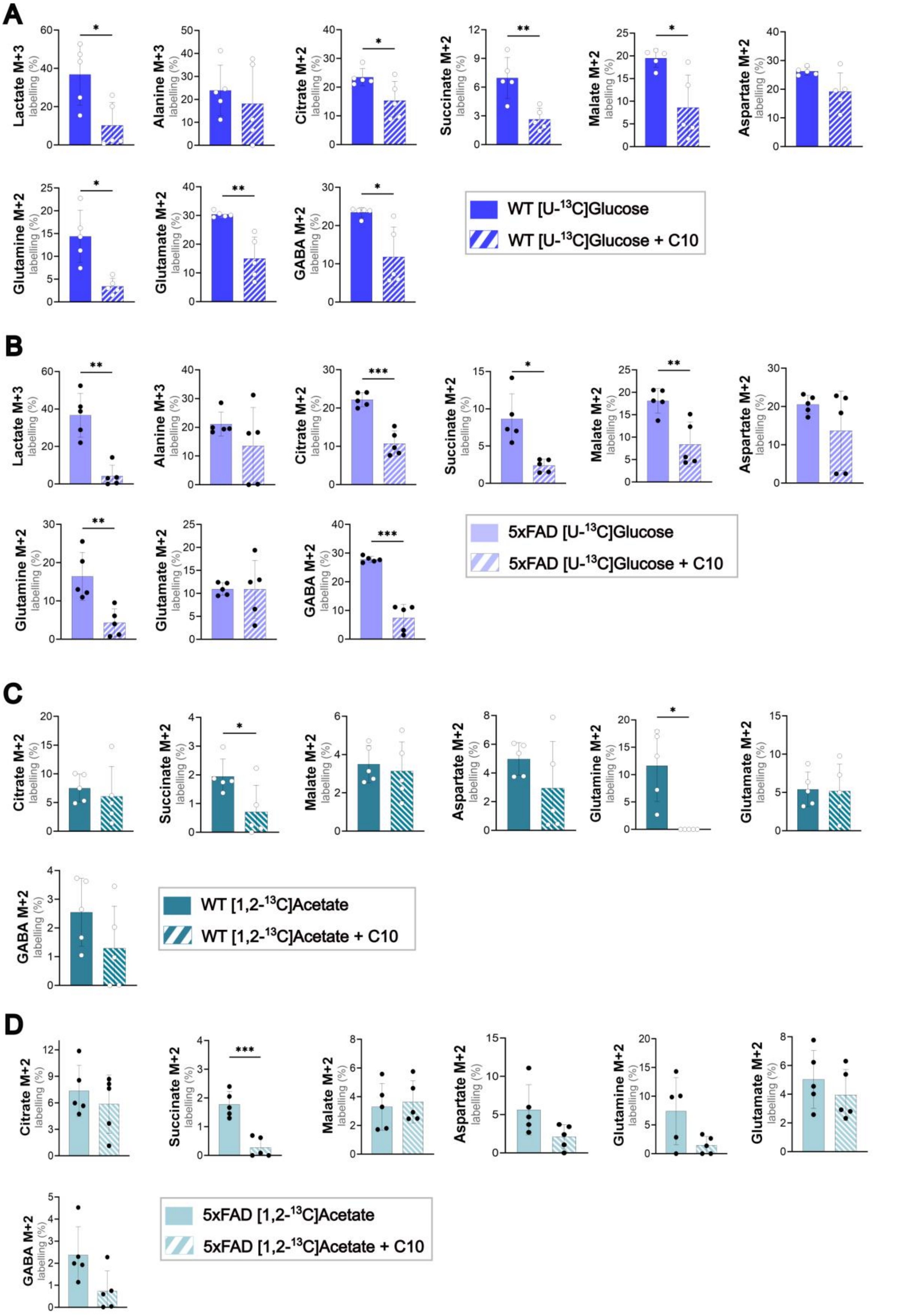

Figure S4. Metabolic competition assay between glucose and C10 in mouse hippocampal slices. (A) Metabolic competition assay between [U-^{13}C]glucose and unlabelled ^{12}C10 in cerebral hippocampal slices of WT mice. **(B)** Metabolic competition assay between [U-^{13}C]glucose and unlabelled ^{12}C10 in

cerebral hippocampal slices of 5xFAD mice. **(C)** Metabolic competition assay between [1,2-^{13}C]acetate and unlabelled ^{12}C10 in cerebral hippocampal slices of WT mice. **(C)** Metabolic competition assay between [1,2-^{13}C]acetate and unlabelled ^{12}C10 in cerebral hippocampal slices of 5xFAD mice. Glycolytic products (M+3) and first turn (M+2) TCA cycle intermediates shown. Concentrations: 5 mM glucose, 5 mM acetate and 0.2 mM C10. Unless incubated with [U-^{13}C]glucose, all incubations were supplemented with 5 mM D-glucose. Mean ± SD, n = 5 from individual animals, Welch's t-test or Mann–Whitney test, *p <0.05, **p <0.01, ***p <0.001.

FULL STATISTICAL REPORT

Table S1 (for Figure 1a)

Type of data: results from ***WT and 5xFAD*** *acutely isolated cerebral* ***cortical*** *slices incubated with* ***[U-13C]glucose*** *WT N= 5*

Nomality: tested by Shapiro-Wilk (S-W) test 5xFAD N= 5

Statistical analysis: Welch's t-test or Mann-Whitney test, two-tailed - unpaired data set

LAC M+3	**Mean**	**SD**	S-W p-value	Normal?	Stat test	*t,df*	*p*	Significant?
**WT**	**33.49**	**8.872**	0.4193	Y				
**5xFAD**	**27.43**	**9.095**	0.6322	Y	Welch	t=1.066, df=7.995	0.3177	N
ALA M+3			S-W p-value	Normal?	Stat test	*t,df*	*p*	Significant?
**WT**	**38.06**	**4.095**	0.5151	Y				
**5xFAD**	**47.17**	**8.077**	0.1451	Y	Welch	t=2.250, df=5.929	0.066	N
CIT M+2			S-W p-value	Normal?	Stat test	*U*	*p*	Significant?
**WT**	**26.3**	**2.403**	0.0187	N				
**5xFAD**	**20.85**	**9.161**	0.0302	N	Mann-Whitney	9	0.5476	N
GLU M+2			S-W p-value	Normal?	Stat test	*t,df*	*p*	Significant?
**WT**	**33.26**	**1.177**	0.375	Y				
**5xFAD**	**30.48**	**4.242**	0.1011	Y	Welch	t=1.412, df=4.612	0.2217	N
GLN M+2			S-W p-value	Normal?	Stat test	*U*	*p*	Significant?
**WT**	**25.73**	**2.752**	0.0135	N				
**5xFAD**	**22.28**	**3.095**	0.4615	Y	Mann-Whitney	4	0.0952	N
GABA M+2			S-W p-value	Normal?	Stat test	*t,df*	*p*	Significant?
**WT**	**27.49**	**0.9657**	0.3242	Y				
**5xFAD**	**26.77**	**0.9115**	0.5872	Y	Welch	t=1.216, df=7.973	0.2589	N
SUC M+2			S-W p-value	Normal?	Stat test	*t,df*	*p*	Significant?
**WT**	**7.731**	**3.186**	0.0751	Y				
**5xFAD**	**5.603**	**4.676**	0.1036	Y	Welch	t=0.8408, df=7.056	0.428	N
MAL M+2			S-W p-value	Normal?	Stat test	*U*	*p*	Significant?
**WT**	**21.92**	**1.801**	0.5519	Y				
**5xFAD**	**17.13**	**9.427**	0.0475	N	Mann-Whitney	9	0.5476	N
ASP M+2			S-W p-value	Normal?	Stat test	*U*	*p*	Significant?
**WT**	**23.69**	**3.216**	0.3192	Y				
**5xFAD**	**20.36**	**6.107**	0.0416	N	Mann-Whitney	7	0.3095	N

Table S2 (for Figure 1b)

Type of data: results from ***WT and 5xFAD*** *acutely isolated cerebral* ***cortical*** *slices incubated with* ***[1,2-13C]acetate***

Nomality: tested by Shapiro-Wilk (S-W) test

Statistical analysis: Welch's t-test or Mann-Whitney test, two-tailed - unpaired data set

WT	*N= 6*
5xFAD	*N= 6*

CIT M+2	**Mean**	**SD**	S-W p-value	Normal?	Stat test	*t,df*	*p*	Significant?
**WT**	6.974	1.384	0.4673	Y				Y
**5xFAD**	4.461	1.639	0.5534	Y	Welch	t=2.870, df=9.726	0.0171	
GLU M+2			S-W p-value	Normal?	Stat test	*t,df*	*p*	Significant?
**WT**	4.641	1.156	0.4892	Y				Y
**5xFAD**	2.741	1.344	0.2617	Y	Welch	t=2.625, df=9.781	0.0258	
GLN M+2			S-W p-value	Normal?	Stat test	*t,df*	*p*	Significant?
**WT**	17.35	2.431	0.4294	Y		t=3.880, df=8.013		Y
**5xFAD**	9.66	4.2	0.5472	Y	Welch		0.0047	
GABA M+2			S-W p-value	Normal?	Stat test	*t,df*	*p*	Significant?
**WT**	4.69	0.8113	0.9208	Y				Y
**5xFAD**	3.279	1.229	0.1758	Y	Welch	t=2.346, df=8.661	0.0447	
SUC M+2			S-W p-value	Normal?	Stat test	*t,df*	*p*	Significant?
**WT**	1.84	0.587	0.3043	Y				Y
**5xFAD**	1.066	0.3593	0.9569	Y	Welch	t=2.754, df=8.285	0.0241	
MAL M+2			S-W p-value	Normal?	Stat test	*t,df*	*p*	Significant?
**WT**	2.94	0.7082	0.9133	Y				Y
**5xFAD**	1.339	0.895	0.1572	Y	Welch	t=3.435, df=9.498	0.0069	
ASP M+2			S-W p-value	Normal?	Stat test	*t,df*	*p*	Significant?
**WT**	2.495	0.6748	0.7673	Y				Y
**5xFAD**	1.158	0.7114	0.1626	Y	Welch	t=3.339, df=9.972	0.0075	

Table S3 (for Figure 2a)

*Type of data: results from **WT and 5xFAD** acutely isolated cerebral **cortical** slices incubated with **[U-13C]C10***
Nomality: tested by Shapiro-Wilk (S-W) test
Statistical analysis: Welch's t-test or Mann-Whitney test, two-tailed- unpaired data set

WT *N= 6*
5xFAD *N= 5*

	Mean	SD	S-W p-value	Normal?	Stat test	*t,df* / *U*		*p*	Significant?
CIT M+2	**Mean**	**SD**	S-W p-value	Normal?	Stat test	*t,df*		*p*	Significant?
**WT**	9.923	2.851	0.4549	Y				0.9845	N
**5xFAD**	9.878	4.26	0.652	Y	Welch	t=0.02015, df=6.788			
GLU M+2			S-W p-value	Normal?	Stat test	*t,df*		*p*	Significant?
**WT**	5.267	1.942	0.1586	Y		t=0.3985, df=7.093		0.702	N
**5xFAD**	5.846	2.721	0.2527	Y	Welch				
GLN M+2			S-W p-value	Normal?	Stat test	*t,df*		*p*	Significant?
**WT**	23.53	2.493	0.9694	Y		t=0.02225, df=6.008		0.983	N
**5xFAD**	23.53	4.482	0.2039	Y	Welch				
GABA M+2			S-W p-value	Normal?	Stat test	*U*		*p*	Significant?
**WT**	4.059	1.418	0.3327	Y			13	0.7922	N
**5xFAD**	4.238	1.684	0.0242	N	Mann-Whitney				
SUC M+2			S-W p-value	Normal?	Stat test	*U*		*p*	Significant?
**WT**	2.101	1.018	0.0225	N			8	0.2468	N
**5xFAD**	1.682	1.127	0.153	Y	Mann-Whitney				
MAL M+2			S-W p-value	Normal?	Stat test	*t,df*		*p*	Significant?
**WT**	4.107	1.734	0.9939	Y		t=0.01356, df=7.513		0.9895	N
**5xFAD**	4.123	2.232	0.4454	Y	Welch				
ASP M+2			S-W p-value	Normal?	Stat test	*U*		*p*	Significant?
**WT**	2.633	1.471	0.0407	N			14	0.9307	N
**5xFAD**	3.153	1.703	0.2053	Y	Mann-Whitney				

Table S4 (for Figure 2b)

Type of data: results from 5xFAD acutely isolated cerebral ***cortical*** *slices incubated with* ***[U-13C]glucose + /-C10***

Nomality: tested by Shapiro-Wilk (S-W) test

Statistical analysis: Welch's t-test or Mann-Whitney test, two-tailed- unpaired data set

WT	*N= 5*
5xFAD	*N= 5*

LAC M+3	**Mean**	**SD**	S-W p-value	Normal?	Stat test	*t,df*		*p*	Significant?
**[U-13C]glucose**	37.0379	6.48158	0.2981	Y		t=2.127, df=5.605		0.0807	N
**[U-13C]glucose+C10**	22.221	14.1612	0.6523	Y	Welch				

ALA M+3			S-W p-value	Normal?	Stat test	*t,df*		*p*	Significant?
**[U-13C]glucose**	29.6003	7.98343	0.2444	Y		t=1.610, df=6.105		0.1576	N
**[U-13C]glucose+C10**	36.1165	4.25784	0.0747	Y	Welch				

CIT M+2			S-W p-value	Normal?	Stat test	*t,df*		*p*	Significant?
**[U-13C]glucose**	27.4059	3.58091	0.8893	Y		t=0.1590, df=4.396		0.8807	N
**[U-13C]glucose+C10**	28.5765	16.0689	0.1713	Y	Welch				

GLU M+2			S-W p-value	Normal?	Stat test	*t,df*		*p*	Significant?
**[U-13C]glucose**	29.5086	1.30467	0.5717	Y		t=5.603, df=5.038		0.0024	Y
**[U-13C]glucose+C10**	19.9328	3.5917	0.7579	Y	Welch				

GLN M+2			S-W p-value	Normal?	Stat test	*U*		*p*	Significant?
**[U-13C]glucose**	19.2847	2.71316	0.0564	Y			0	0.0079	Y
**[U-13C]glucose+C10**	4.20585	2.64358	0.0241	N					

GABA M+2			S-W p-value	Normal?	Stat test	*t,df*		*p*	Significant?
**[U-13C]glucose**	25.5026	2.77	0.4686	Y		t=2.530, df=7.977		0.0353	Y
**[U-13C]glucose+C10**	21.1838	2.62566	0.9253	Y	Welch				

SUC M+2			S-W p-value	Normal?	Stat test	*t,df*		*p*	Significant?
**[U-13C]glucose**	7.87058	2.03955	0.858	Y		t=3.208, df=7.974		0.0125	Y
**[U-13C]glucose+C10**	3.60891	2.15905	0.2891	Y	Welch				

MAL M+2			S-W p-value	Normal?	Stat test	*t,df*		*p*	Significant?
**[U-13C]glucose**	17.6806	2.93691	0.6091	Y		t=2.822, df=6.715		0.0269	Y
**[U-13C]glucose+C10**	10.6919	4.69512	0.1356	Y	Welch				

ASP M+2			S-W p-value	Normal?	Stat test	*t,df*		*p*	Significant?
**[U-13C]glucose**	19.4426	3.71418	0.128	Y		t=3.968, df=7.259		0.005	Y
**[U-13C]glucose+C10**	11.3286	2.6676	0.8459	Y	Welch				

Table S5 (for Figure 2c)

Type of data: results from ***5xFAD*** *acutely isolated cerebral* ***cortical*** *slices incubated with* ***[1,2-13C]acetate + /-C10***

Nomality: tested by Shapiro-Wilk (S-W) test

Statistical analysis: Welch's t-test or Mann-Whitney test, two-tailed- unpaired data set

WT	*N= 5*
5xFAD	*N= 5*

CIT M+2	**Mean**	**SD**	S-W p-value	Normal?	Stat test	*t,df*		*p*	Significant?
**[1,2-13C]acetate**	6.41697	2.73839	0.9916	Y		t=0.9433, df=7.986		0.3732	N
**[1,2-13C]acetate+C10**	8.08617	2.85639	0.8032	Y	Welch				

GLU M+2			S-W p-value	Normal?	Stat test	*t,df*		*p*	Significant?
**[1,2-13C]acetate**	4.56572	1.89321	0.6226	Y		t=3.112, df=5.994		0.0208	Y
**[1,2-13C]acetate+C10**	1.60023	0.97822	0.8615	Y	Welch				

GLN M+2			S-W p-value	Normal?	Stat test	*U*		*p*	Significant?
**[1,2-13C]acetate**	14.5919	4.07337	0.9947	Y			0	0.0079	Y
**[1,2-13C]acetate+C10**	0.14856	0.33218	0.0001	N	Mann-Whitney				

GABA M+2			S-W p-value	Normal?	Stat test	*t,df*		*p*	Significant?
**[1,2-13C]acetate**	3.83561	1.29062	0.5915	Y		t=4.534, df=5.221		0.0056	Y
**[1,2-13C]acetate+C10**	1.0214	0.51023	0.1254	Y	Welch				

SUC M+2			S-W p-value	Normal?	Stat test	*U*		*p*	Significant?
**[1,2-13C]acetate**	2.55432	0.60652	0.3008	Y			0	0.0079	Y
**[1,2-13C]acetate+C10**	0.28746	0.3571	0.0079	N	Mann-Whitney				

MAL M+2			S-W p-value	Normal?	Stat test	*t,df*		*p*	Significant?
**[1,2-13C]acetate**	2.5175	1.31114	0.7837	Y		t=2.631, df=4.782		0.0486	Y
**[1,2-13C]acetate+C10**	0.90074	0.41194	0.4477	Y	Welch				

ASP M+2			S-W p-value	Normal?	Stat test	*t,df*		*p*	Significant?
**[1,2-13C]acetate**	2.1838	1.25878	0.9025	Y		t=3.100, df=4.853		0.0279	Y
**[1,2-13C]acetate+C10**	0.34671	0.41352	0.1133	Y	Welch				

Table S6 (for Figure 3)

Type of data: results from hiPSC derived astrocytes carrying AD mutations incubated with [U-13C]glucose +/- C10 unpaired data set

Nomality: tested by Shapiro-Wilk (S-W) test

Statistical analysis: Welch's t-test or Mann-Whitney test, two-tailed- unpaired data set *N= 5-6*

	K3P53		PSEN-1 A280E		APP London		APP Swedish	
	[U13-C]glucose	[U13-C]glucose+C10	[U13-C]glucose	[U13-C]glucose+C10	[U13-C]glucose	[U13-C]glucose+C10	[U13-C]glucose	[U13-C]glucose+C10
**LAC M+3**								
Mean	7.123	9.185	5.782	7.174	10.65	14.86	10.13	13.11
SD	1.775	2.787	0.9562	2.675	2.593	3.704	4.278	2.47
S-W p-value	0.7354	0.2925	0.3961	0.3918	0.7013	0.553	0.2364	0.9965
Normal?	Y	Y	Y	Y	Y	Y	Y	Y
Stat test	Welch		Welch		Welch		Welch	
t,df or U	t=1.528, df=8.484		t=1.096, df=5.006		t=2.281, df=8.952		t=1.445, df=8.167	
p	0.1628		0.3231		0.0486		0.1858	
Significant?	N		N		Y		N	
**ALA M+3**								
Mean	32.15	20.24	32.69	23.55	33.92	24.44	29.69	23.41
SD	5.426	6.543	2.97	2.033	4.648	4.071	8.102	3.619
S-W p-value	0.9302	0.3293	0.3547	0.0341	0.2676	0.9863	0.3598	0.6174
Normal?	Y	Y	Y	N	Y	Y	Y	Y
Stat test	Welch		Mann-Whitney		Welch		Welch	
t,df or U	t=3.433, df=9.669		0		t=3.757, df=9.829		t=1.706, df=7.167	
p	0.0067		0.0079		0.0039		0.1308	
Significant?	Y		Y		Y		N	
**CIT M+2**								
Mean	24.11	26.61	25.16	24.98	13.93	22.09	20.35	21.36
SD	3.348	4.999	4.087	5.588	5.068	5.576	6.629	6.037
S-W p-value	0.4076	0.5949	0.7461	0.6816	0.3027	0.7557	0.5278	0.3396
Normal?	Y	Y	Y	Y	Y	Y	Y	Y
Stat test	Welch		Welch		Welch		Welch	
t,df or U	t=1.016, df=8.735		t=0.05556, df=7.327		t=2.652, df=9.910		t=0.2653, df=8.894	
p	0.3368		0.9572		0.0244		0.7969	
Significant?	N		N		Y		N	
**α-KG M+2**								
Mean	8.917	15.04	14.94	17.02	8.89	11.46	8.653	13.71
SD	4.775	2.136	2.685	3.128	4.504	3.17	2.053	0.5586
S-W p-value	0.0685	0.5101	0.2924	0.0226	0.2459	0.9803	0.1879	0.0207
Normal?	Y	Y	Y	N	Y	Y	Y	N
Stat test	Welch		Mann-Whitney		Welch		Mann-Whitney	
t,df or U	t=2.867, df=6.924		8		t=1.141, df=8.978		0	
p	0.0244		0.4206		0.2832		0.0043	
Significant?	Y		N		N		Y	
**GLU M+2**								
Mean	24.54	23.27	26.03	25.34	29.06	27.59	23.27	21.64
SD	1.858	1.944	1.136	1.237	1.372	1.007	0.5832	1.101
S-W p-value	0.2583	0.4107	0.0234	0.0312	0.3987	0.1063	0.813	0.3557
Normal?	Y	Y	N	N	Y	Y	Y	Y
Stat test	Welch		Mann-Whitney		Welch		Welch	
t,df or U	t=1.152, df=9.980		8		t=2.116, df=9.178		t=2.979, df=5.835	
p	0.2761		0.4206		0.0629		0.0255	
Significant?	N		N		N		Y	
**GLN M+2**								
Mean	4.073	6.45	5.016	6.284	3.782	5.378	5.453	6.146
SD	2.663	1.685	2.656	0.8646	1.74	1.04	3.213	1.716
S-W p-value	0.7758	0.125	0.4473	0.7337	0.6023	0.0036	0.763	0.845
Normal?	Y	Y	Y	Y	Y	N	Y	Y
Stat test	Welch		Welch		Mann-Whitney		Welch	
t,df or U	t=1.847, df=8.452		t=1.015, df=4.839		10		t=0.4558, df=7.858	
p	0.0999		0.3581		0.2403		0.6609	
Significant?	N		N		N		N	
**SUC M+2**								
Mean	0.2367	1.26	2.09	1.954	0.3717	0.7217	1.627	2.122
SD	0.3646	0.8184	0.7493	0.5477	0.3864	0.6121	1.345	0.8479
S-W p-value	0.0166	0.6885	0.9091	0.4633	0.1332	0.2808	0.8282	0.4843
Normal?	N	Y	Y	Y	Y	Y	Y	Y
Stat test	Mann-Whitney		Welch		Welch		Welch	
t,df or U	5.5		t=0.3277, df=7.325		t=1.184, df=8.439		t=0.7422, df=8.491	
p	0.0433		0.7523		0.2686		0.478	
Significant?	Y		N		N		N	
**MAL M+2**								
Mean	9.382	9.468	11.08	10.62	11.83	10.9	9.403	9.408
SD	1.192	0.7514	0.8741	0.9018	1.597	1.178	1.753	1.082
S-W p-value	0.3884	0.3858	0.3946	0.3188	0.8953	0.583	0.2388	0.5492
Normal?	Y	Y	Y	Y	Y	Y	Y	Y
Stat test	Welch		Welch		Welch		Welch	
t,df or U	t=0.1507, df=8.433		t=0.8047, df=7.992		t=1.150, df=9.200		t=0.005401, df=8.417	
p	0.8838		0.4442		0.2792		0.9958	
Significant?	N		N		N		N	
**ASP M+2**								
Mean	9.642	8.717	10.54	10.58	9.893	9.637	8.17	8.138
SD	0.7063	1.013	0.5997	0.5107	1.787	1.103	0.7122	0.5691
S-W p-value	0.681	0.8779	0.4234	0.0418	0.4073	0.9522	0.2591	0.4349
Normal?	Y	Y	Y	N	Y	Y	Y	Y
Stat test	Welch		Mann-Whitney		Welch		Welch	
t,df or U	t=1.835, df=8.932		12		t=0.2994, df=8.329		t=0.08281, df=8.996	
p	0.1		>0.9999		0.772		0.9358	
Significant?	N		N		N		N	

Table S7 (for Figure S1a)

*Type of data: results from **WT and 5xFAD** acutely isolated cerebral **hippocampal** slices incubated with **[U-13C]glucose*** — *WT N= 5*

Nomality: tested by Shapiro-Wilk (S-W) test — *5xFAD N= 5*

Statistical analysis: Welch's t-test or Mann-Whitney test, two-tailed - unpaired data set

LAC M+3	**Mean**	**SD**	S-W p-value	Normal?	Stat test	*t,df*		*p*	Significant?
**WT**	30.1037	12.6248	0.1208	Y		t=2.408, df=6.063		0.0523	N
**5xFAD**	14.7339	6.65416	0.8531	Y	Welch				
ALA M+3			S-W p-value	Normal?	Stat test	*t,df*		*p*	Significant?
**WT**	44.0806	7.34264	0.3551	Y		t=3.428, df=7.552		0.0098	Y
**5xFAD**	25.7746	9.41442	0.8087	Y	Welch				
CIT M+2			S-W p-value	Normal?	Stat test	*U*		*p*	Significant?
**WT**	26.0446	2.28963	0.7377	Y			7	0.3095	N
**5xFAD**	19.9197	10.9402	0.0103	N	Mann-Whitney				
GLU M+2			S-W p-value	Normal?	Stat test	*U*		*p*	Significant?
**WT**	32.9298	1.91679	0.0272	N			2	0.0317	Y
**5xFAD**	30.2258	1.13202	0.004	N	Mann-Whitney				
GLN M+2			S-W p-value	Normal?	Stat test	*U*		*p*	Significant?
**WT**	8.38422	2.8759	0.0006	N			8	0.4206	N
**5xFAD**	11.9095	6.60108	0.4075	Y	Mann-Whitney				
GABA M+2			S-W p-value	Normal?	Stat test	*U*		*p*	Significant?
**WT**	28.0142	2.15744	0.9832	Y			9	0.5476	N
**5xFAD**	22.3212	12.3281	0.0008	N	Mann-Whitney				
SUC M+2			S-W p-value	Normal?	Stat test	*t,df*		*p*	Significant?
**WT**	5.45079	1.73131	0.0613	Y		t=0.1894, df=4.719		0.8577	N
**5xFAD**	5.60392	0.52127	0.2727	Y	Welch				
MAL M+2			S-W p-value	Normal?	Stat test	*t,df*		*p*	Significant?
**WT**	20.6982	1.31683	0.4337	Y		t=2.454, df=7.999		0.0397	Y
**5xFAD**	18.6436	1.33085	0.9711	Y	Welch				
ASP M+2			S-W p-value	Normal?	Stat test	*U*		*p*	Significant?
**WT**	23.7838	1.39031	0.6401	Y			3	0.0556	N
**5xFAD**	17.3131	9.78532	0.0146	N	Mann-Whitney				

Table S8 (for Figure S1b)

Type of data: results from ***WT and 5xFAD*** *acutely isolated cerebral* ***hippocampal*** *slices incubated with* ***[1,2-13C]acetate***

Nomality: tested by Shapiro-Wilk (S-W) test

Statistical analysis: Welch's t-test or Mann-Whitney test, two-tailed - unpaired data set

WT	*N= 5*
5xFAD	*N= 6*

CIT M+2	**Mean**	**SD**	S-W p-value	Normal?	Stat test	*t,df*	*p*	Significant?
**WT**	6.22566	2.35526	0.5677	Y		t=1.412, df=7.079	0.2003	N
**5xFAD**	7.99929	1.67573	0.1409	Y	Welch			
GLU M+2			S-W p-value	Normal?	Stat test	*t,df*	*p*	Significant?
**WT**	4.55487	0.96983	0.3178	Y		t=0.5600, df=7.036	0.5929	N
**5xFAD**	4.84373	0.68392	0.3591	Y	Welch			
GLN M+2			S-W p-value	Normal?	Stat test	*t,df*	*p*	Significant?
**WT**	8.29384	5.9918	0.8648	Y		t=0.6943, df=5.514	0.5157	N
**5xFAD**	10.3242	2.86967	0.5812	Y	Welch			
GABA M+2			S-W p-value	Normal?	Stat test	*t,df*	*p*	Significant?
**WT**	4.8855	0.7257	0.9889	Y		t=0.1914, df=8.967	0.8525	N
**5xFAD**	4.79534	0.83682	0.3492	Y	Welch			
SUC M+2			S-W p-value	Normal?	Stat test	*t,df*	*p*	Significant?
**WT**	0.80259	0.33817	0.2242	Y		t=1.155, df=7.809	0.2822	N
**5xFAD**	1.0212	0.27871	0.7042	Y	Welch			
MAL M+2			S-W p-value	Normal?	Stat test	*t,df*	*p*	Significant?
**WT**	3.25764	0.90193	0.7458	Y		t=0.2334, df=7.595	0.8216	N
**5xFAD**	3.37367	0.71199	0.1761	Y	Welch			
ASP M+2			S-W p-value	Normal?	Stat test	*t,df*	*p*	Significant?
**WT**	2.48965	0.52619	0.7842	Y		t=0.2062, df=8.785	0.8414	N
**5xFAD**	2.55677	0.55128	0.7317	Y	Welch			

Table S9 (for Figure S2a)

Type of data: results from ***WT*** *and* ***5xFAD*** *acutely isolated cerebral* ***hippocampal slices*** *incubated with* ***[U-13C]C10*** — *WT N= 6*, *5xFAD N= 6*

Nomality: tested by Shapiro-Wilk (S-W) test

Statistical analysis: Welch's t-test or Mann-Whitney test, two-tailed- unpaired data set

CIT M+2	**Mean**	**SD**	S-W p-value	Normal?	Stat test	*t,df*	*p*	Significant?
**WT**	6.86923	2.72911	0.9593	Y		t=1.937, df=9.433	0.0833	N
**5xFAD**	9.60383	2.1247	0.6824	Y	Welch			

GLU M+2			S-W p-value	Normal?	Stat test	*U*	*p*	Significant?
**WT**	3.53363	1.37182	0.5067	Y		11	0.3095	N
**5xFAD**	4.35176	0.72613	0.0288	N	Mann-Whitney			

GLN M+2			S-W p-value	Normal?	Stat test	*t,df*	*p*	Significant?
**WT**	17.0764	1.73627	0.538	Y		t=0.1317, df=5.964	0.8996	N
**5xFAD**	16.7631	5.56519	0.1894	Y	Welch			

GABA M+2			S-W p-value	Normal?	Stat test	*t,df*	*p*	Significant?
**WT**	3.4195	0.86973	0.203			t=1.671, df=10	0.1257	N
**5xFAD**	4.09898	0.48577	0.9353		Welch			

SUC M+2			S-W p-value	Normal?	Stat test	*t,df*	*p*	Significant?
**WT**	0.68518	0.20395	0.1593	Y		t=2.476, df=9.137	0.0348	Y
**5xFAD**	1.03554	0.28019	0.6514	Y	Welch			

MAL M+2			S-W p-value	Normal?	Stat test	*t,df*	*p*	Significant?
**WT**	4.00858	1.02768	0.8516	Y		t=1.720, df=9.948	0.1164	N
**5xFAD**	4.99411	0.95605	0.5461	Y	Welch			

ASP M+2			S-W p-value	Normal?	Stat test	*t,df*	*p*	Significant?
**WT**	1.97057	0.52968	0.532	Y		t=2.125, df=9.991	0.0595	N
**5xFAD**	2.61084	0.51382	0.5147	Y	Welch			

Table S10 (for Figure S2b)

Type of data: results from ***WT*** *acutely isolated cerebral* ***cortical*** *slices incubated with* ***[U-13C]glucose + /-C10*** WT *N= 5*

Nomality: tested by Shapiro-Wilk (S-W) test 5xFAD *N= 4*

Statistical analysis: Welch's t-test or Mann-Whitney test, two-tailed- unpaired data set

LAC M+3	**Mean**	**SD**	S-W p-value	Normal?	Stat test	*t,df*	*p*	Significant?
**[U-13C]glucose**	43.2068	8.52793	0.6384	Y		t=1.339, df=6.272	0.2269	N
**[U-13C]glucose+C10**	35.1582	9.28707	0.2662	Y	Welch			

ALA M+3			S-W p-value	Normal?	Stat test	*t,df*	*p*	Significant?
**[U-13C]glucose**	33.0582	7.34921	0.3033	Y		t=3.077, df=6.831	0.0184	Y
**[U-13C]glucose+C10**	47.4518	6.65763	0.2661	Y	Welch			

CIT M+2			S-W p-value	Normal?	Stat test	*t,df*	*p*	Significant?
**[U-13C]glucose**	27.8515	1.84662	0.9137	Y		t=1.286, df=3.079	0.2865	N
**[U-13C]glucose+C10**	37.1958	14.4332	0.0846	Y	Welch			

GLU M+2			S-W p-value	Normal?	Stat test	*t,df*	*p*	Significant?
**[U-13C]glucose**	31.3762	1.80474	0.5781	Y		t=1.361, df=3.316	0.2586	N
**[U-13C]glucose+C10**	26.4567	7.04587	0.3831	Y	Welch			

GLN M+2			S-W p-value	Normal?	Stat test	*t,df*	*p*	Significant?
**[U-13C]glucose**	24.7605	2.59663	0.6201	Y		t=16.76, df=5.237	<0.0001	Y
**[U-13C]glucose+C10**	3.76149	0.94247	0.8937	Y	Welch			

GABA M+2			S-W p-value	Normal?	Stat test	*U*	*p*	Significant?
**[U-13C]glucose**	27.7515	1.30027	0.0499	N		7	0.5556	N
**[U-13C]glucose+C10**	24.5029	5.42486	0.4122	Y	Mann-Whitney			

SUC M+2			S-W p-value	Normal?	Stat test	*t,df*	*p*	Significant?
**[U-13C]glucose**	9.29181	1.88881	0.1908	Y		t=3.306, df=6.374	0.0149	Y
**[U-13C]glucose+C10**	4.96372	2.00046	0.5553	Y	Welch			

MAL M+2			S-W p-value	Normal?	Stat test	*t,df*	*p*	Significant?
**[U-13C]glucose**	20.9544	2.40344	0.8137	Y		t=2.710, df=7	0.0302	Y
**[U-13C]glucose+C10**	15.1074	4.05478	0.0862	Y	Welch			

ASP M+2			S-W p-value	Normal?	Stat test	*t,df*	*p*	Significant?
**[U-13C]glucose**	22.903	2.88298	0.9178	Y		t=3.668, df=6.219	0.0098	Y
**[U-13C]glucose+C10**	15.3889	3.18431	0.3561	Y	Welch			

Table S11 (for Figure S2c)

Type of data: results from ***WT*** *acutely isolated cerebral* ***cortical*** *slices incubated with* ***[1,2-13C]acetate + /-C10***

WT *N= 5*
5xFAD *N= 4*

Nomality: tested by Shapiro-Wilk (S-W) test

Statistical analysis: Welch's t-test or Mann-Whitney test, two-tailed- unpaired data set

CIT M+2	**Mean**	**SD**	S-W p-value	Normal?	Stat test	*t,df*	*p*	Significant?
**[1,2-13C]acetate**	4.17724	2.42398	0.3631	Y		t=0.06503, df=4.412	0.951	N
**[1,2-13C]acetate+C10**	4.33794	4.44106	0.6008	Y	Welch			
GLU M+2			S-W p-value	Normal?	Stat test	*t,df*	*p*	Significant?
**[1,2-13C]acetate**	3.25375	1.64021	0.1592	Y		t=0.7816, df=5.931	0.4645	N
**[1,2-13C]acetate+C10**	2.29995	1.95053	0.298	Y	Welch			
GLN M+2			S-W p-value	Normal?	Stat test	*t,df*	*p*	Significant?
**[1,2-13C]acetate**	12.1355	4.61956	0.8948	Y		t=5.250, df=4.590	0.0043	Y
**[1,2-13C]acetate+C10**	0.88907	1.13327	0.1493	Y	Welch			
GABA M+2			S-W p-value	Normal?	Stat test	*t,df*	*p*	Significant?
**[1,2-13C]acetate**	3.03658	1.00946	0.8462	Y		t=2.984, df=6.997	0.0204	Y
**[1,2-13C]acetate+C10**	1.23959	0.79714	0.5236	Y	Welch			
SUC M+2			S-W p-value	Normal?	Stat test	*t,df*	*p*	Significant?
**[1,2-13C]acetate**	2.18208	0.81721	0.7025	Y		t=4.100, df=6.785	0.0049	Y
**[1,2-13C]acetate+C10**	0.3371	0.52506	0.0552	Y	Welch			
MAL M+2			S-W p-value	Normal?	Stat test	*t,df*	*p*	Significant?
**[1,2-13C]acetate**	1.53092	1.06092	0.0603	Y		t=0.4030, df=6.980	0.699	N
**[1,2-13C]acetate+C10**	1.28373	0.77767	0.7332	Y	Welch			
ASP M+2			S-W p-value	Normal?	Stat test	*U*	*p*	Significant?
**[1,2-13C]acetate**	1.32924	1.00527	0.0812	Y		3	0.1111	Y
**[1,2-13C]acetate+C10**	0.60103	0.54237	0.0341	N	Mann-Whitney			

Table S12 (for Figure S3a)

Type of data: results from ***WT*** *acutely isolated cerebral* ***hippocampal*** *slices incubated with* ***[U-13C]glucose + /-C10*** WT *N= 5*

Nomality: tested by Shapiro-Wilk (S-W) test 5xFAD *N= 5*

Statistical analysis: Welch's t-test or Mann-Whitney test, two-tailed- unpaired data set

	Mean	SD	S-W p-value	Normal?	Stat test	*t,df*		*p*	Significant?
LAC M+3									
**[U-13C]glucose**	36.8118	15.9319	0.4361	Y		t=2.981, df=7.407		0.0192	Y
**[U-13C]glucose+C10**	10.2908	11.9093	0.0654	Y	Welch				
ALA M+3			S-W p-value	Normal?	Stat test	*t,df*		*p*	Significant?
**[U-13C]glucose**	23.879	11.0022	0.6068	Y		t=0.6292, df=6.808		0.5498	N
**[U-13C]glucose+C10**	18.1382	17.1822	0.2358	Y	Welch				
CIT M+2			S-W p-value	Normal?	Stat test	*t,df*		*p*	Significant?
**[U-13C]glucose**	23.5118	2.98375	0.0582	Y		t=2.522, df=5.570		0.0482	Y
**[U-13C]glucose+C10**	15.3424	6.59924	0.5219	Y	Welch				
GLU M+2			S-W p-value	Normal?	Stat test	*t,df*		*p*	Significant?
**[U-13C]glucose**	30.4253	1.04147	0.402	Y		t=4.558, df=4.155		0.0095	Y
**[U-13C]glucose+C10**	15.0285	7.48167	0.6235	Y	Welch				
GLN M+2			S-W p-value	Normal?	Stat test	*t,df*		*p*	Significant?
**[U-13C]glucose**	14.3999	5.73614	0.9592	Y		t=4.091, df=4.701		0.0107	Y
**[U-13C]glucose+C10**	3.45061	1.70458	0.8285	Y	Welch				
GABA M+2			S-W p-value	Normal?	Stat test	*U*		*p*	Significant?
**[U-13C]glucose**	23.4953	1.26761	0.0037	N			1	0.0159	Y
**[U-13C]glucose+C10**	11.8297	7.77441	0.1035	Y	Mann-Whitney				
SUC M+2			S-W p-value	Normal?	Stat test	*t,df*		*p*	Significant?
**[U-13C]glucose**	6.96042	2.14565	0.8528	Y		t=3.959, df=6.103		0.0072	Y
**[U-13C]glucose+C10**	2.65498	1.14377	0.7321	Y	Welch				
MAL M+2			S-W p-value	Normal?	Stat test	*t,df*		*p*	Significant?
**[U-13C]glucose**	19.5059	2.04402	0.2064	Y		t=3.276, df=4.648		0.0245	Y
**[U-13C]glucose+C10**	8.60244	7.15614	0.3222	Y	Welch				
ASP M+2			S-W p-value	Normal?	Stat test	*t,df*		*p*	Significant?
**[U-13C]glucose**	26.2436	1.32403	0.8078	Y		t=2.383, df=4.333		0.0708	N
**[U-13C]glucose+C10**	19.195	6.48055	0.7991	Y	Welch				

Table S13 (for Figure S3b)

Type of data: results from ***5xFAD*** *acutely isolated cerebral* ***hippocampal*** *slices incubated with* ***[U-13C]glucose + /-C10*** WT *N= 5*
Nomality: tested by Shapiro-Wilk (S-W) test 5xFAD *N= 5*
Statistical analysis: Welch's t-test or Mann-Whitney test, two-tailed- unpaired data set

LAC M+3	**Mean**	**SD**	S-W p-value	Normal?	Stat test	*t,df*		*p*	Significant?
**[U-13C]glucose**	36.6725	11.7113	0.9344	Y		t=5.555, df=5.830		0.0016	Y
**[U-13C]glucose+C10**	4.24679	5.7631	0.0519	Y	Welch				
ALA M+3			S-W p-value	Normal?	Stat test	*U*		*p*	Significant?
**[U-13C]glucose**	21.1633	4.17867	0.0066	N			5	0.1508	N
**[U-13C]glucose+C10**	13.5252	13.3802	0.3163	Y	Mann-Whitney				
CIT M+2			S-W p-value	Normal?	Stat test	*t,df*		*p*	Significant?
**[U-13C]glucose**	22.1919	1.83701	0.3796	Y		t=6.861, df=6.322		0.0004	Y
**[U-13C]glucose+C10**	10.7438	3.24779	0.4531	Y	Welch				
GLU M+2			S-W p-value	Normal?	Stat test	*t,df*		*p*	Significant?
**[U-13C]glucose**	10.955	1.3587	0.2971	Y		t=0.02651, df=4.367		0.98	N
**[U-13C]glucose+C10**	10.8782	6.33409	0.8563	Y	Welch				
GLN M+2			S-W p-value	Normal?	Stat test	*t,df*		*p*	Significant?
**[U-13C]glucose**	16.422	6.28368	0.2276	Y		t=3.724, df=6.411		0.0087	Y
**[U-13C]glucose+C10**	4.32917	3.63864	0.5984	Y	Welch				
GABA M+2			S-W p-value	Normal?	Stat test	*t,df*		*p*	Significant?
**[U-13C]glucose**	27.8339	1.03648	0.8445	Y		t=9.416, df=4.382		0.0005	Y
**[U-13C]glucose+C10**	7.42104	4.73556	0.0595	Y	Welch				
SUC M+2			S-W p-value	Normal?	Stat test	*t,df*		*p*	Significant?
**[U-13C]glucose**	8.63889	3.36386	0.3049	Y		t=4.028, df=4.519		0.0123	Y
**[U-13C]glucose+C10**	2.3855	0.8582	0.154	Y	Welch				
MAL M+2			S-W p-value	Normal?	Stat test	*t,df*		*p*	Significant?
**[U-13C]glucose**	18.1203	2.76482	0.2477	Y		t=3.809, df=6.250		0.0082	Y
**[U-13C]glucose+C10**	8.41458	4.9819	0.1164	Y	Welch				
ASP M+2			S-W p-value	Normal?	Stat test	*t,df*		*p*	Significant?
**[U-13C]glucose**	20.5323	2.42388	0.8437	Y		t=1.443, df=4.435		0.2158	N
**[U-13C]glucose+C10**	13.6588	10.3742	0.0536	Y	Welch				

Table S14 (for Figure S3c)

Type of data: results from ***WT*** *acutely isolated cerebral* ***hippocampal*** *slices incubated with* ***[1,2-13C]acetate + /-C10***

Nomality: tested by Shapiro-Wilk (S-W) test

Statistical analysis: Welch's t-test or Mann-Whitney test, two-tailed- unpaired data set

WT	*N= 5*
5xFAD	*N= 5*

CIT M+2	**Mean**	**SD**	S-W p-value	Normal?	Stat test	*t,df*	*p*	Significant?
**[1,2-13C]acetate**	7.51666	2.43753	0.2523	Y		t=0.5604, df=5.685	0.5966	N
**[1,2-13C]acetate+C10**	6.08043	5.18694	0.2405	Y	Welch			

GLU M+2			S-W p-value	Normal?	Stat test	*t,df*	*p*	Significant?
**[1,2-13C]acetate**	5.42007	2.26445	0.6147	Y		t=0.1255, df=8	0.9032	N
**[1,2-13C]acetate+C10**	5.18467	3.52963	0.9905	Y	Welch			

GLN M+2			S-W p-value	Normal?	Stat test	*t,df*	*p*	Significant?
**[1,2-13C]acetate**	11.6298	6.51943	0.4734	Y		t=3.989, df=4.000	0.0163	Y
**[1,2-13C]acetate+C10**	0	0			Welch			

GABA M+2			S-W p-value	Normal?	Stat test	*t,df*	*p*	Significant?
**[1,2-13C]acetate**	2.54799	1.18468	0.463			t=1.485, df=7.659	0.1776	N
**[1,2-13C]acetate+C10**	1.29582	1.46752	0.4005		Welch			

SUC M+2			S-W p-value	Normal?	Stat test	*t,df*	*p*	Significant?
**[1,2-13C]acetate**	1.94963	0.60471	0.1213	Y		t=2.502, df=6.885	0.0414	Y
**[1,2-13C]acetate+C10**	0.71207	0.9263	0.1244	Y	Welch			

MAL M+2			S-W p-value	Normal?	Stat test	*t,df*	*p*	Significant?
**[1,2-13C]acetate**	3.50242	0.97349	0.2836	Y		t=0.4487, df=6.791	0.6676	N
**[1,2-13C]acetate+C10**	3.13908	1.52674	0.7595	Y	Welch			

ASP M+2			S-W p-value	Normal?	Stat test	*t,df*	*p*	Significant?
**[1,2-13C]acetate**	4.97685	1.14061	0.112	Y		t=1.316, df=4.968	0.2456	N
**[1,2-13C]acetate+C10**	2.94696	3.25503	0.1771	Y	Welch			

Table S15 (for Figure S3d)

Type of data: results from ***5xFAD*** *acutely isolated cerebral* ***hippocampal*** *slices incubated with* ***[1,2-13C]acetate + /-C10***

Nomality: tested by Shapiro-Wilk (S-W) test

Statistical analysis: Welch's t-test or Mann-Whitney test, two-tailed- unpaired data set

WT	*N= 5*
5xFAD	*N= 5*

CIT M+2	**Mean**	**SD**	S-W p-value	Normal?	Stat test	*t,df*	*p*	Significant?
**[1,2-13C]acetate**	7.35503	2.8922	0.3333	Y		t=0.7674, df=7.868	0.4653	N
**[1,2-13C]acetate+C10**	5.85067	3.2942	0.2161	Y	Welch			

GLU M+2			S-W p-value	Normal?	Stat test	*t,df*	*p*	Significant?
**[1,2-13C]acetate**	5.0335	2.01459	0.9328	Y		t=0.8997, df=7.876	0.3949	N
**[1,2-13C]acetate+C10**	3.95295	1.77561	0.1832	Y	Welch			

GLN M+2			S-W p-value	Normal?	Stat test	*t,df*	*p*	Significant?
**[1,2-13C]acetate**	7.41805	5.84025	0.5871			t=2.199, df=4.545	0.0846	N
**[1,2-13C]acetate+C10**	1.48079	1.52828	0.345		Welch			

GABA M+2			S-W p-value	Normal?	Stat test	*t,df*	*p*	Significant?
**[1,2-13C]acetate**	2.3774	1.27477	0.1696	Y		t=2.332, df=7.279	0.0511	N
**[1,2-13C]acetate+C10**	0.73786	0.92027	0.1311	Y	Welch			

SUC M+2			S-W p-value	Normal?	Stat test	*t,df*	*p*	Significant?
**[1,2-13C]acetate**	1.77655	0.45032	0.7411	Y		t=5.886, df=7.515	0.0005	Y
**[1,2-13C]acetate+C10**	0.27958	0.34734	0.0508	Y	Welch			

MAL M+2			S-W p-value	Normal?	Stat test	*t,df*	*p*	Significant?
**[1,2-13C]acetate**	3.30381	1.62934	0.529	Y		t=0.3508, df=7.914	0.7349	N
**[1,2-13C]acetate+C10**	3.64784	1.46788	0.1738	Y	Welch			

ASP M+2			S-W p-value	Normal?	Stat test	*t,df*	*p*	Significant?
**[1,2-13C]acetate**	5.6375	3.28225	0.2738	Y		t=2.158, df=5.755	0.0762	N
**[1,2-13C]acetate+C10**	2.12219	1.57771	0.5946	Y	Welch			
